# The influence of age-dependent susceptibility on RSV transmission dynamics and immunisation population-level impact

**DOI:** 10.1186/s12916-026-04776-1

**Published:** 2026-03-11

**Authors:** Chenkai Zhao, Yuhe Zhang, Richard Osei-Yeboah, Xiao Li, You Li, Xin Wang, Harish Nair

**Affiliations:** 1 Department of Epidemiology, National Vaccine Innovation Platform, School of Public Health, Nanjing Medical University, Nanjing, China; 2 Department of Biostatistics, National Vaccine Innovation Platform, School of Public Health, Nanjing Medical University, Nanjing, China; 3 Centre for Global Health, Usher Institute, University of Edinburgh, Edinburgh, UK; 4 Centre for Health Economics Research and Modelling Infectious Diseases, Vaccine and Infectious Disease Institute, University of Antwerp, Antwerp, Belgium; 5 Key Laboratory of Public Health Safety and Emergency Prevention and Control Technology of Higher Education Institutions in Jiangsu Province, Nanjing Medical University, Nanjing, China; 6 Changzhou Third People’s Hospital, Changzhou Medical Centre, Nanjing Medical University, Changzhou, China

**Keywords:** Respiratory syncytial virus, Transmission, Age-dependent susceptibility, Immunisation

## Abstract

**Background:**

Respiratory syncytial virus (RSV) causes substantial disease burden worldwide, disproportionately affecting infants and young children. Higher susceptibility to RSV infections in young children, interacting with age-varying contact patterns and risk of disease progression, could drive the age-related variations in disease burden, though the extent of the susceptibility differences, and their influence on transmission dynamics and immunisation impact remains unclear.

**Methods:**

We developed an age-structured deterministic transmission model that integrated virological surveillance data, hospitalisations, and infection rates from the UK. We estimated age-dependent susceptibility coefficients for the 0– < 5 years, 5–59 year and ≥ 60 years by integrating a profile likelihood approach with Markov Chain Monte Carlo (MCMC)-based calibration. Models were fitted to weekly RSV-positive cases in Scotland and infection rate estimates for the UK population. Age-specific infection-hospitalisation ratios (IHRs) were derived by combining modelled infections with RSV-associated hospitalisations. We evaluated multiple paediatric immunisation scenarios by estimating infections and hospitalisations averted, and the number of individuals needed to immunise (NNI) to prevent one RSV hospitalisation at varying coverage and efficacy.

**Results:**

We estimated a susceptibility coefficient of 0.38 (95% CI 0.36–0.38) for 5–59 years, and 0.38 (0.20–0.40) for ≥ 60 years, relative to those under 5 years. The overall annual infection rate was 51.8%, with peaked in children aged 12–23 months (71.1%) and 0–2 months (63.7%) using the best-fitting model, showing a substantial shift in the age distribution compared to the base model. IHRs showed a U-shaped distribution, with the highest rates in infants aged 0–2 months and adults aged 75 and above. This model also projected a greater impact from immunisation programmes compared to the base model. For instance, an infant immunisation programme with 80% coverage and 80% efficacy against hospitalisation was projected to prevent 15.2% of hospitalisations, compared to 8.9% in the base model. Broader programmes, such as targeting 0–4-year-olds, resulted in larger reductions in hospitalisations (27.1%), while NNI increased as the programme expanded.

**Conclusions:**

The increased RSV susceptibility in children under 5 years drives higher baseline transmission rates and disease burden in this subgroup, thereby influencing immunisation programme impact and efficiency.

**Supplementary Information:**

The online version contains supplementary material available at 10.1186/s12916-026-04776-1.

## Background

Respiratory syncytial virus (RSV) is a leading cause of lower respiratory tract infections, causing substantial hospitalisations each year, disproportionately affecting young children and older adults [1, 2]. In the UK, annual RSV-associated RTI hospitalisation rate showed substantial age-dependent variations, peaking at 40–90 cases per 1000 infants under 6 months of age while dropping below 0.3 cases per 1000 individuals aged 18–64 years [3, 4]. The variations in RSV disease burden across age groups could be partly driven by the age-dependent transmission patterns. Empirical data suggest differential age-dependent transmission patterns between respiratory viruses, including RSV, SARS-CoV-2 and influenza, even when circulating within the same populations [5–7]. These distinct patterns suggest that beyond mixing patterns, age-dependent variations in susceptibility to these infections likely play a significant role in shaping their transmission dynamics and disease burden. However, the exact influence of age-dependent susceptibility in shaping RSV transmission dynamics has not yet been clearly characterised.

Various types of research indicate increased susceptibility to RSV infections in young children compared to older age groups. A South African study found a higher RSV attack rate among household contacts under 5 years compared to those 5 years and older. Another Philippine household study showed that 52% of household members under 5 years were infected within a week after exposure to RSV, compared to 19% for those 5 years or older. Furthermore, serological research also showed low neutralising antibody titers against RSV during acute infection among infants and young children [8, 9], indicating inadequate protective immunity in this population.

Significant progress has been made in RSV prophylaxis development in recent years. A monoclonal antibody (nirsevimab) and a maternal RSV pre-fusion F protein vaccine (RSVpreF) have been approved for use in a number of countries, primarily in high-income settings, to protect infants from RSV infections. Maternal RSVpreF vaccination administered during pregnancy showed 69% efficacy within 180 days after birth in reducing severe RSV-associated lower respiratory infections in a phase 3 trial [10]. Nirsevimab, recommended for infants and high-risk children, showed 82–90% effectiveness in preventing RSV-associated hospitalisations among children younger than 24 months in real-world studies across multiple countries [11–13]. Additionally, several RSV vaccine candidates targeting children younger than 5 years are currently in development, with most candidates undergoing evaluation in phase I and II clinical trials [14]. Despite significant advances in RSV preventive interventions for infants and young children, their population-level impact may be substantially modulated by age-varying susceptibility patterns that shape transmission dynamics and age-dependent disease burden. However, the cascading effects of age-dependent susceptibility remain poorly characterised.

Accurate characterisation, inference and prediction of RSV transmission dynamics could be obscured by incomplete ascertainment of RSV infections due to a lack of systematic viral testing, and under-representation of asymptomatic and mild infections in routine surveillance data. To address the uncertainties, we developed a modelling framework that incorporates multiple data sources, including RSV virological surveillance data, hospitalisations and infection rates, to evaluate the extent of age-dependent variations in susceptibility, and its influence on RSV transmission dynamics and immunisation programme population-level impact. Our analysis specifically focuses on immunisation programmes targeting the age subgroups showing increased RSV susceptibility.

## Methods

### Transmission model

We developed an age-structured, deterministic compartmental transmission model, grouping individuals into four epidemiological states: maternally protected (M), susceptible (S), infectious (I) and recovered (R) with temporal immunity, while allowing for possible repeat infections in the same and subsequent seasons. We assumed that all newborns had temporary maternal antibodies against RSV infections (M) for $1/{\omega }_{m}$ days after birth, before transitioning into the susceptible state. Infected individuals transition to the infectious state ($1/\gamma$ days), followed by temporary immunity ($1/{\omega }_{Inf}$ days) before becoming susceptible to reinfections. Our model assumed shorter infectious periods for repeat infections, with a scaling factor (${\sigma }_{1}$) for the second infection and a factor (${\sigma }_{1}\cdot {\sigma }_{2}$) for the third and subsequent infections, similar to previous literature (Additional file 1: Section 1) [15–22].

Individuals entered and left the modelled populations through ageing, birth and mortality. We categorised individuals into 11 age groups: 0–2 months, 3–5 months, 6–11 months, 12–23 months, 2–4 years, 5–19 years, 20–59 years, 60–64 years, 65–69 years, 70–74 years and 75 years and above. Individuals transitioned between age groups at rates inversely proportional to each age band’s duration. The initial population age structure was sourced from 2018 Scottish demographic data [16], with birth rates parameterised using 2009–2019 Scottish averages [17]. The mortality rate was set equal to the birth rate to maintain a stable population size. Using the contact matrix for the UK arm of the POLYMOD study [23], and the contact matrix reported by another UK contact survey [24], we calculated age-specific contact rates with adjustments for changes in population age distribution between the original contact survey and our study period (Additional file 1: Section 2) [23–25].

We included seasonal forcing in the force of infection to capture temporal variations in RSV transmissibility. The seasonal transmission rate was modelled using a normal distribution function centred at the transmissibility peak [15, 26], resulting in a well-defined single-peak epidemic season with low baseline transmission outside the season in a year. Based on observed UK surveillance data, we set the transmissibility peak to week 47, corresponding to the reported peak in RSV activity (Additional file 1: Section 3) [27].

### Data sources, model parameters, model calibration and estimation of age-dependent susceptibility coefficients

We incorporated multiple data sources. The primary data source was weekly RSV test-positive cases from all laboratories in Scotland, sourced from the Electronic Communication of Surveillance in Scotland system [28]. We assumed test-positive cases were a proportion of the total infections with constant age-specific reporting rates during the study period. We included data from July 2017 to April 2023, and defined each epidemic year as the period from July to June of the following year. The 2017/18–2019/20 RSV surveillance data representing typical RSV transmission dynamics, were used for characterising age-dependent susceptibility and evaluating immunisation impact. Following the disruption of RSV circulation during the COVID-19 pandemic, we compared the RSV time-varying reproduction number (Rt) between the pandemic and pre-pandemic levels to explore transmissibility changes [29]. Additionally, we incorporated the annual average RSV-associated respiratory tract infection (RSV-RTI) hospitalisation rates across ten age groups (0–2 months, 3–5 months, 6–11 months, 12–35 months, 3–4 years, 5–17 years, 18–64 years, 65–74 years, 75 years and above), estimated based on age-specific temporal associations between RTI hospitalisations and RSV test-positive cases during 2010–2016 [4]. We mapped the age-specific hospitalisation rates to the closest age groups, i.e. the hospitalisation rate among individuals aged 12–35 months for 12–23 months, 3–4 years for 2–4 years, 5–17 years for 5–19 years, 18–64 years for 20–59 years, and the average hospitalisation rate in 65–74 years for those aged 60 years and above (Additional file 2: Table S1). Furthermore, we included the RSV infection rate for infants under 1 year from a UK sero-epidemiological birth cohort study [30].

We modelled age-specific susceptibility for three age groups, i.e. individuals under 5 years, 5–59 years, and 60 years and above. Children under 5 years were selected as the reference, with a coefficient of 1.0, reflecting their potentially higher susceptibility compared to the older age groups. The susceptibility coefficients for 5–59 and ≥ 60 years groups were estimated using a two-stage strategy that integrated a profile likelihood approach with Markov Chain Monte Carlo (MCMC) calibration. In the first stage, we performed calibration against weekly RSV-positive case data to optimise all other parameters across a grid of susceptibility coefficients using MCMC. The grid was designed to include 176 value pairs for the two susceptibility coefficients, spanning 0.3–0.6 for the 5–59 years group and 0.2–0.4 for the ≥ 60 years group, both in increments of 0.02. The grouping and ranges were chosen based on age-specific RSV infection risk from a cohort study [5]. For each fixed pair of susceptibility coefficients, we optimised all other parameters using MCMC. Each simulation began on January 1, 1999, with ten infectious individuals seeded in each age group, and reached equilibrium prior to data fitting. We calibrated the product of simulated age-specific RSV infections and reporting rates to weekly test-positive cases from 2017/18 to 2019/20 using a negative binomial likelihood joint with a Dirichlet-multinomial likelihood for the age distribution of cases before and after December (Additional file 1: Section 4) [31]. We used non-informative priors for all free parameters. In total, 14 model parameters were estimated in this stage, including three transmission parameters and 11 age-specific reporting rates of RSV infections. After 10,000 burn-in iterations, we ran 20,000 iterations and sampled every tenth iteration to reduce autocorrelation. In the second stage, the likelihood of each model output obtained from the first stage, was calculated against the RSV infection rate data. The 95% confidence intervals (CIs) of the susceptibility coefficients were estimated based on likelihood ratios [32]. We estimated infection-hospitalisation ratios (IHRs) as the ratio of estimated RSV hospitalisations to model-estimated infections for each age group, using parameters from the best-fitting model. To characterise the influence of age-dependent susceptibility on RSV transmission dynamics, we implemented a homogeneous susceptibility scenario, calibrated independently, as a baseline control (referred as the base scenario). Additionally, we conducted a sensitivity analysis using a duration of 190 days for natural immunity, selected based on previous literature, to assess the robustness of our results to this parameter [19].

#### Impact of RSV immunisation programmes

We stratified individuals into the immunised (the intervention efficacy against RSV infections × coverage × 100%) and the remaining unimmunised groups. We assumed that immunisation-induced protection was temporary lasting for $1/{\omega }_{\mathrm{immu}}$ days (Table S3), before the immunised individuals became susceptible. Based on literature, we modelled a larger intervention efficacy against RSV hospitalisations than infections by assuming a proportion ([the efficacy against RSV hospitalisations – the efficacy against RSV infections] × coverage × 100%) of immunised individuals could be infected with RSV but not progress to severe diseases requiring hospitalisations [33, 34]. A remaining proportion (the efficacy against RSV infections × coverage × 100%) of immunised individuals were not susceptible to RSV infections during the protection period (Additional file 1: Section 5) [10, 11, 35–38].

Our primary objective was to investigate the influence of age-varying susceptibility on the population-level impact of immunisation. To this aim, we modelled a simplified implementation plan where the target intervention coverage was reached, and all immunised individuals were protected since 1 st September of a calendar year. We assessed various immunisation implementation scenarios by different target age groups (0–12 months, 0–24 months and 0–4 years), immunisation coverage (60% and 80%) and efficacy (60%, 70% and 80% against RSV infections, and 70%, 80% and 90% against hospitalisations). We assessed the population-level impact of these immunisation implementation alternatives using the best-fitting model, compared to the homogeneous-susceptibility base model using two analytical strategies: (1) applying the infection-hospitalisation ratios derived from the best-fitting model to both models (fixed-IHR approach); (2) using model-specific infection-hospitalisation ratios (fixed-rate approach) derived by simulations and hospitalisation rates. For each immunisation implementation strategy and model, we estimated the annual reductions in RSV infections and hospitalisations, overall and stratified by immunisation status, and the proportion decrease in peak weekly RSV hospitalisations during the first year after the implementation for each alternative, compared with a no-immunisation counterfactual scenario. We estimated the number of individuals needed to immunise (NNI) to prevent one RSV-RTI hospitalisation, as the ratio of total number of immunised individuals to the total averted hospitalisations. We reported the median estimates and 95% Credible intervals (CrIs) for the IHRs and immunisation impacts based on 1000 samples from the posterior distribution.

#### Statistical software and code availability

Data analyses and visualisations were conducted using R software (version 4.3.2). The transmission model was programmed in C + +. All codes used in this analysis are available in the GitHub repository (https://github.com/ChenkaiZhao-086/RSV_trans_model).

#### Role of the funding source

The funders of the study had no role in study design, data collection, data analysis, data interpretation, writing of the manuscript or the decision to submit for publication.

## Results

**Fig. 1:**
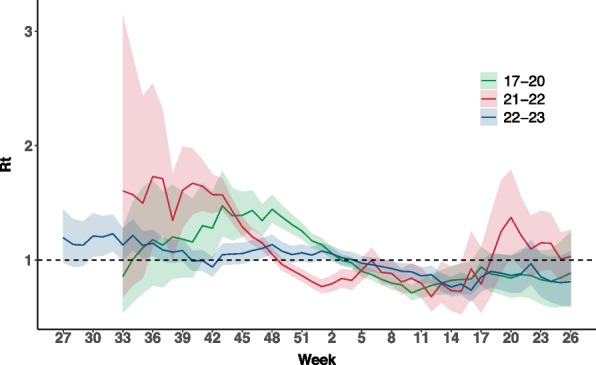
Time-varying reproduction number (Rt) estimates between 2017/18 and 2022/23 seasons. The solid line represents the median of time-varying reproduction number (Rt) from 2017 to 2023, and the shaded ribbon represents the 95% credible interval

RSV showed relatively consistent seasonality in Scotland during the 2017/18 to 2019/20 season, with test-positive cases peaking between calendar week 43 and 48 each year. In the 2020/21 season, RSV circulation remained at low levels due to COVID-19 control measures. During the 2021/22 season, RSV case counts started rising earlier than in pre-pandemic years, peaking in week 39. The estimated peak time-varying reproduction number (Rt) during the epidemic was 1.73 (95% CrIs 1.11–2.31), representing a 20% increase over the 2017/18–2019/20 average peak transmissibility (1.44, 1.32–1.57). Subsequently, a second, smaller outbreak occurred from late 2021/22 through 2022/23, with the peak Rt of 1.37 (1.02–1.79) (Fig. 1).

**Fig. 2:**
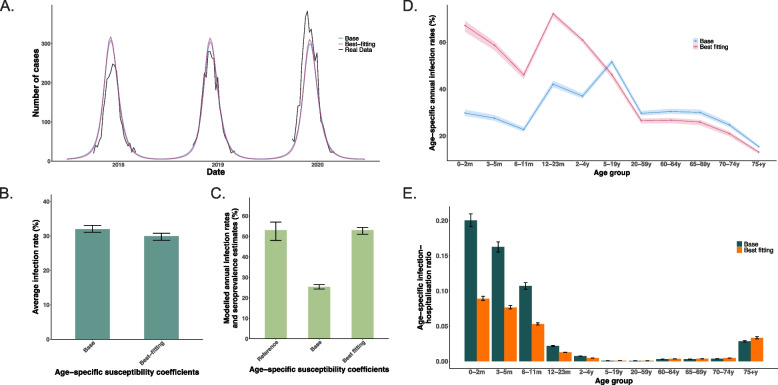
Comparison of the best-fitting and the base model. The susceptibility coefficient was 1.0 for 0–4 years old, 5–59 years old and 60 years and above in the base model, and was 1.0, 0.38 and 0.38 in the best-fitting model. **A** Model fits to RSV test-positive cases. Black lines show reported RSV test-positive cases; lines in other varying colours show the posterior median estimates from different models. **B** Model-estimated annual RSV infection rates for all ages combined. **C** The reported RSV infection rates for infants younger than 1 year according to a UK seroprevalence study (i.e. the reference), and model-estimated RSV infection rates for infants. **D** Model-estimated age-specific RSV infection rates. **E** Age-specific infection-hospitalisation ratio estimates using best-fitting model versus the base model

**Fig. 3:**
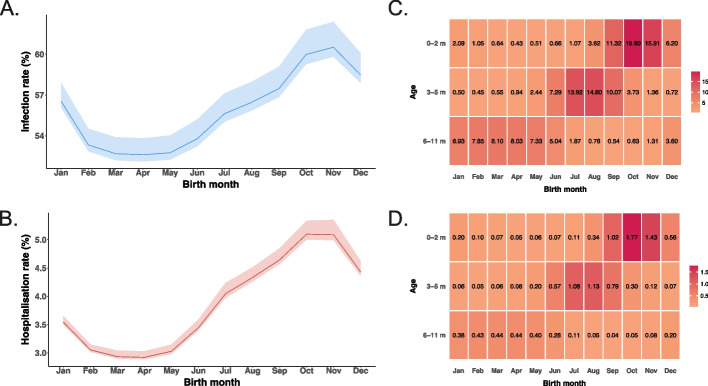
Age-specific infection and hospitalisation rates by birth month in infants aged 0–11 months. This figure shows infant infection rates and hospitalisation rates across different age groups stratified by birth month. **A** Age-specific infection rates for infants in the first year of life, by birth month. Lines indicate the medians and shaded areas indicate 95% credible intervals. **B** Age-specific hospitalisation rates for infants in the first year of life, by birth month. **C** Monthly infection rates, by age and birth month. **D** Monthly hospitalisation rates, by age and birth month

We estimated a susceptibility coefficient of 0.38 (95% CI 0.36–0.38) for 5–59 years, and 0.38 (0.20–0.40) for ≥ 60 years, relative to those under 5 years (Additional file 2: Table S2-S3). Using the best-fitting model, we estimated that the annual average RSV infection rate ranged between 46.0% and 71.1% among individuals under 19 years, from 20.9% to 26.6% in those aged 20–74 years, and further decreased to 13.0% (12.3–13.5) in those aged 75 years and above (Fig. 2). Infants born in November (corresponding to the epidemic peak month) had the highest RSV infection rate (60.5%, 95% CrI 59.8–62.4) in their first year, followed by those born in October and December, while those born in October and November had the highest hospitalisation rate (5.1% 5.0–5.3%) (Fig. 3).The estimated infection-hospitalisation ratios (IHRs) firstly declined from infants under 1 year old (0.089, 95% CrI 0.086–0.093) to a nadir in 20–59 years (0.00072, 0.00070–0.00075), then increased from adults aged 60–64 years (0.0036, 0.0035–0.0038) to those 75 years and above (0.033, 0.032–0.035) (Fig. 2E). In the sensitivity analysis using a shorter immunity duration, the annual infection rate among infants was similar to the main analysis (52.7% vs 51.9%).

While both the best-fitting and base model with different susceptibility assumptions reproduced the observed dynamics of RSV test-positive cases during the 2017/18–2019/20 seasons (Fig. 2A), they produced different transmissibility estimates and caused age-shifting in RSV transmission dynamics (Fig. 2B–D, Additional file 2: Fig. S1-S2). Compared to the homogeneous-susceptibility base scenario, a reduction in susceptibility among the ≥ 5 years groups (the best-fitting model) was associated with a substantially higher estimated average transmission rate. The overall RSV infection rate declined modestly from 32.0% in the base scenario to 27.0% in the best-fitting model (Fig. 2B). Age-specific RSV infection rates peaked among population aged 5–19 years (51.6%), followed by the 12–23 months group (42.1%) in the base scenario (Fig. 2D). In contrast, infection rates increased substantially among children under 5 years using the best-fitting model, with the highest infection rates found in children aged 12–23 months, followed by infants aged 0–2 months. We estimated higher IHRs for the ≥ 65 years group, but lower estimates for children under 5 years old using the best-fitting model, compared to the base scenario. The IHRs for children under 5 years reduced by 39.2–55.6% compared to the base model, while the estimates for individuals 5 years old and above were slightly higher.

**Fig. 4:**
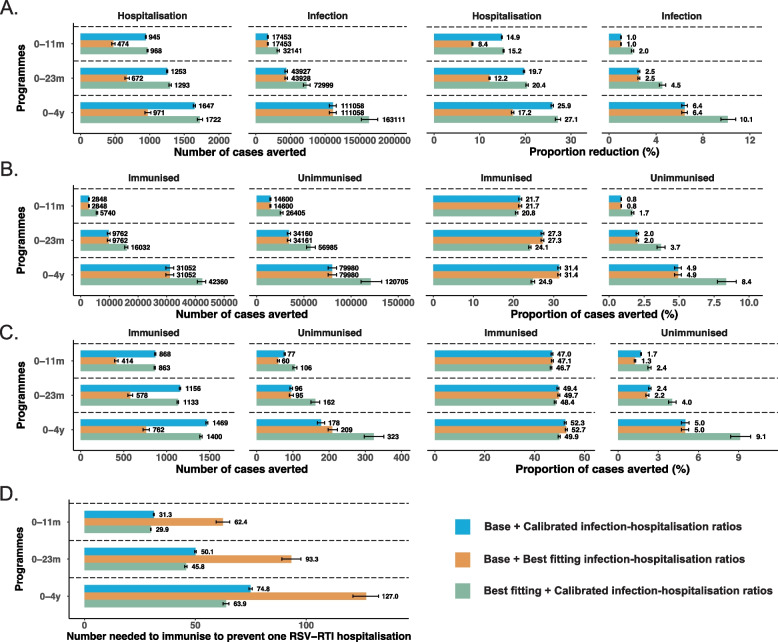
Population-level impact of immunisation programmes using the best-fitting model versus the base model. This figure illustrates the simulated population-level impact of RSV immunisation programmes for infants and children under 5 using the best-fitting model versus the base model. The susceptibility coefficient was 1.0, 1.0 and 1.0 for 0–4 years old, 5–59 years old and 60 years and above in the base model, and 1.0, 0.38 and 0.38 in the best-fitting model; immunisation efficacy: 70%/80% denotes a 70% efficacy against RSV infections and a 80% efficacy against hospitalisations; coverage: 80%; target age groups: 0–12 months old, 0–24 months old, and 0–4 years old. Calibrated IHRs: infection-hospitalisation ratios derived from either the best-fitting model or the base model. Best-fitting IHRs: the infection-hospitalisation ratios derived from the best-fitting model. **A** The number and proportion of averted infections and hospitalisations in all ages combined. **B** The number and proportion of averted infections in immunised and unimmunised populations. **C** The number and proportion of averted hospitalisations in immunised and unimmunised populations. **D** The number of people needed to be immunised to prevent one RSV hospitalisation

Applying the IHRs derived from the best-fitting model to both models, the best-fitting model consistently demonstrated greater population-level impact and efficiency in reducing both RSV infections and hospitalisations compared to the base model across all the alternatives, at 80% coverage and 70%/80% efficacy (against RSV infections/hospitalisations) (Fig. 4A–D). For instance, the best-fitting model estimated that an infant (0–11 months) immunisation programme prevented 32,141 (30,765–33,825) infections and 968 (961–975) hospitalisations in the first year after implementation, reducing overall RSV infections and hospitalisations by 2.0% (1.9–2.1) and 15.2% (15.1–15.4), respectively, for all ages combined. In contrast, the base model showed 17,453 infections (16,897–18,031) and 474 (451–498) hospitalisations prevented, reducing overall RSV infections by 1.0% (1.0–1.0) and RSV hospitalisations by 8.4% (8.3–8.5) (Fig. 4A). The best-fitting model estimated greater absolute reductions in RSV hospitalisations among the immunised population compared to the base model, but projected a smaller proportion decrease due to the substantially higher baseline incidence. The best-fitting model estimated an NNI of 29.9 (29.8–30.1) for the infant immunisation programme to prevent one RSV-RTI hospitalisation, while the base model showed an NNI of 62.4 (59.4–65.5) (Fig. 4D). Larger differences in the prevented RSV infections and hospitalisations for all ages combined were found between the two models as immunisation programmes expanded to children under 2 and 5 years old (Fig. 4).

When using model-specific infection-hospitalisation ratios derived by calibration, the best-fitting model showed greater reductions in both case counts and proportion decrease of overall RSV infections compared to the base model, while the two models showed comparable reductions in hospitalisations (Fig. 4). Nevertheless, the best-fitting model estimated a lower NNI across the immunisation programmes, with the differences becoming larger as programmes expanded.

**Fig. 5:**
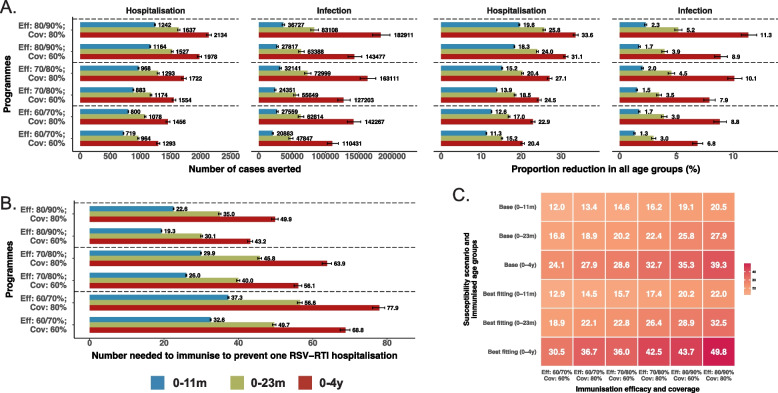
Impact of infant and child RSV immunisation on population-level disease burden under the best-fitting model. This figure illustrates the simulated population-level impact of RSV immunisation programmes for infants and children under 5 years across varying efficacy and coverage, using the best-fitting model. Eff 60%/70% denotes a 60% efficacy against RSV infections and a 70% efficacy against hospitalisations (same for Eff 70%/80% and Eff 80%/90%). Cov: coverage rates. **A** The number and proportion of averted infections and hospitalisations in all ages combined at varying efficacy and coverage. **B** The number of people needs to be immunised to prevent one RSV hospitalisation. **C** Percentage reductions in the peak of RSV hospitalisations during the first year after the implementation of each programme

Using the best-fitting model, expanding from infants to children under 2 and 5 years and increasing the intervention efficacy from 60%/70% (against infections/hospitalisations) to 80%/90% yielded greater reductions in RSV infections and hospitalisations than raising immunisation coverage from 60 to 80% (Fig. 5). Expanding immunisation programmes to children under 2 and 5 years averted an additional 3.9% and 9.1% RSV-associated hospitalisations at 60% coverage and 60%/70% efficacy respectively, compared to the infant immunisation programme (15.2% [15.0–15.4] and 20.4% [20.0–20.8] vs 11.3% [11.2–11.4]; Fig. 5A). For an infant immunisation programme, increasing efficacy from 60%/70% to 80%/90% averted an additional 7.0% RSV hospitalisations at 60% coverage. On the other hand, higher intervention efficacy lowered the NNI to prevent one RSV hospitalisation, whereas expanding coverage and target groups increased the NNI (Fig. 5B, Additional file 2: Fig. S11). The impacts of immunisation programmes under other susceptibility scenarios are in Additional file 2: Fig. S3-S11.

## Discussion

Using a transmission modelling framework incorporating multiple data sources, i.e. RSV virological surveillance data, hospitalisations and infection rates, we estimated a susceptibility coefficient of 0.38 (95% CI 0.36–0.38) for 5–59 years, and 0.38 (0.20–0.40) for ≥ 60 years, relative to those under 5 years. This susceptibility profile led to substantially greater population-level reductions in RSV infections and hospitalisations, and higher programme efficiency when targeting infants and children under 5 years old, compared to the homogeneous-susceptibility base model at identical IHRs. To our knowledge, this is the first study to systematically assess age-dependent susceptibility to RSV infections while accounting for contact patterns, and to evaluate its implications for transmission dynamics, and the population-level impact and efficiency of immunisation programmes targeting infants and children under 5 years old.

While both the best-fitting and base models could reconstruct the observed dynamics of RSV test positives, they produced divergent results of infection age distributions and IHRs. In particular, the best-fitting model projected higher annual RSV infection rates among children under 5 years that declined with increasing age, similar to the age distribution patterns observed in a South African household study [5]. Among children under 5 years of age, RSV incidence was higher in children aged 12–23 months than in infants aged 0–11 months, consistent with results from another Australian birth cohort [39]. Furthermore, our estimated IHR for children under 2 years (3.5%) was similar to the results from an English birth cohort study that showed an IHR of 3.0–3.5% for children aged 0–23 months, supporting the validity of our model [30]. We found U-shaped age distributions of IHRs, with the lowest IHR found in adults aged 20–59 years. This pattern may reflect both age-related differences in disease progression to severe infections and healthcare seeking behaviour.

The substantial differences in projected immunisation programme impact between the best-fitting and the base models at identical IHRs, show the critical influence of increased RSV susceptibility in children under 5 years on immunisation impact for this age group. While several previous RSV modelling studies adopted fixed IHRs [15, 40], others estimated IHRs from hospitalisation rates [33, 41, 42]. Both approaches should theoretically align when the model accurately reflects RSV transmission dynamics and when true IHRs and hospitalisation rates are known. However, our results have shown divergence when age-dependent susceptibility is misspecified; while the fixed-rate approach provides more robust estimates of averted hospitalisations than the fixed-IHR approach, it could systematically bias IHRs and the impact of transmission control measures in such scenarios.

The UK has implemented RSV immunisation programmes for pregnant persons to protect infants particularly those younger than 6 months, and for individuals aged 75 to 79 years old since September 2024 [43], to reduce the burden of severe RSV diseases. While children younger than 5 years have lower IHRs than young infants, they have been considered key drivers of the community RSV transmission due to their frequent contacts that facilitate RSV transmission within the group and across the broader community. Our analysis incorporated both direct and indirect protection of immunisation programmes, and quantified the population-level impact of programmes targeting infants and childcare centre-attending children (12–23-month-olds, 2–4-year-olds). We parameterised the model using realistic efficacy and coverage estimates for RSV immunisation, with efficacy estimates comparable to nirsevimab trial results in infant populations and coverage rates aligning with the 60% uptake achieved by the UK RSV vaccination programme in older adults by March 2025 [12, 37, 44]. The 0–4–year–old programme was most effective in reducing hospitalisations, followed by the 0–23–month–old programme and the infant programme, while the programme efficiency decreased when the eligible age groups were expanded. These results provide key information for future cost-effectiveness analysis of paediatric RSV immunisation programmes, and support evidence-based policy making for RSV immunisation programmes.

We acknowledge several limitations. Firstly, we estimated age-dependent susceptibility coefficients using a two-stage strategy integrating profile likelihood method with MCMC calibration, which allowed us to address practical identifiability challenges between parameters such as the baseline transmission rate and susceptibility coefficients, and to estimate the age-dependent susceptibility coefficients with 95% confidence intervals. Moreover, as an alternative hypothesis, the homogeneous-susceptibility base model substantially underestimated the infant infection rate, providing additional support for our results. Secondly, we parameterised maternal immunity using the population-average approach for all newborns, consistent with sero-epidemiological data showing > 90% seroprevalence of RSV IgG at birth [30, 45]. While this approach does not account for variations in natural maternal immunity due to differences in maternal exposure history, it has been shown in previous research to properly capture aggregated RSV dynamics in young infants [46, 47]. Thirdly, while our model captured the annual average RSV transmission dynamics, it did not account for potential between-year variations in transmissibility or reporting rates. Fourthly, we assumed homogeneous contact rates within several age groups (0–11 months, 1–4 years and 70 years and above), and potential variations in mixing patterns within these groups could affect the results. We assumed a uniform timing for immunisation implementation, which may overestimate true population-level impacts in scenarios where real-world rollout fails to achieve target coverage by RSV season onset due to implementation challenges. On the other hand, less invasive vaccines such as intranasal vaccines may show improved acceptance in young children, potentially leading to higher uptake rates [48, 49]. Our analysis did not account for protective effects of maternal vaccination for pregnant women, resulting in an underestimation of the overall immunisation impact. Furthermore, the lack of granular hospitalisation data precludes our further stratification for the 0– < 1 month group or accurate evaluation of strategies like maternal vaccination targeting this youngest group. Future research with detailed age-specific data would be essential to address this question. We assumed that RSV interventions provided protection against all RSV infections regardless of symptoms. This assumption was supported by a US cohort study in older adults, where vaccinated individuals had 61.2 (95% confidence interval 16.9–163.2) RSV infections per 1,000 person-years, including symptomatic and asymptomatic infections, compared to 165.8 (88.0–287.0) in unvaccinated individuals [50]. Additionally, we did not account for the potential impact of palivizumab; however, the low uptake rate among eligible populations [51] in the UK suggests that this omission is unlikely to substantially influence our results. Future research could evaluate how the increased susceptibility of young children impacts cost-effectiveness of RSV immunisation strategies, and explore how this age-dependent susceptibility profile compares to that of other viruses. A robust comparison between viruses will require more granular data on the population-level transmission of other relevant viruses beyond influenza and RSV.

## Conclusions

Our study assesses age-dependent susceptibility to RSV infections, and its influence on transmission dynamics and immunisation programme impact. These findings help better understand and predict RSV transmission dynamics and disease burden, and provide a framework for evaluating future intervention strategies.

## Supplementary Information

Additional file 1. Supplementary methods text.

Additional file 2: Figure S1. Trace plots of the parameters for the base scenario. Figure S2. Trace plots of the parameters for the best-fitting model. Figure S3. Population-wide averted infections and hospitalisations under the base model. Figure S4. Population-wide averted infections and hospitalisations under best fitting model. Figure S5. Population-wide averted infections and hospitalisations under the base model with infection-hospitalisation ratios derived from best-fitting model. Figure S6. Population-wide proportion of averted infections and hospitalisations in all ages combined under the base model. Figure S7. Population-wide proportion of averted infections and hospitalisations in all age combined under best-fitting model. Figure S8. Population-wide proportion of averted infections and hospitalisations in all age combined under the base model with infection-hospitalisation ratios derived from best-fitting model. Figure S9. The number of people need to be immunised to prevent one RSV hospitalisation under the base model. Figure S10. The number of people need to be immunised to prevent one RSV hospitalisation under best-fitting model. Figure S11. The number of people needed to be immunised to prevent one RSV hospitalisation under the base model with infection-hospitalisation ratios derived from best-fitting model. Table S1. Hospitalisation rate from previous research. Table S2. Estimated infection rate for infant and log-likelihood under different combinations of age-specific parameters. Table S3. Parameter estimates from different models.

## Data Availability

The data used in the study are derived from publicly available sources. All codes used in this analysis are available in the GitHub repository (https:/github.com/ChenkaiZhao-086/RSV_trans_model).

## Abbreviations

CrIs: Credible intervals
IHRs: Infection-hospitalisation ratios
MCMC: Markov chain Monte Carlo
NNI: Number of individuals needed to immunise
RSV: Respiratory syncytial virus
RSVpreF: RSV pre-fusion F protein vaccine
RSV-RTI: RSV-associated respiratory tract infection
