# Supplementary material for: The influence of age-dependent susceptibility on RSV transmission dynamics and immunisation population-level impact
Source: BMC Med. 2026 Mar 11;24:254. doi: 10.1186/s12916-026-04776-1 (PMC13088621; doi:10.1186/s12916-026-04776-1)
Supplement: Supplementary file 1 — Additional file 1. Supplementary methods text. [file 12916_2026_4776_MOESM1_ESM.docx]

**Additional file 1 for The influence of age-dependent susceptibility on RSV transmission dynamics and immunisation population-level impact**

**Methods**

Chenkai Zhao, Yuhe Zhang, Richard Osei-Yeboah, Xiao Li, You Li, Xin Wang, Harish Nair

Contents

[RSV model structure 2](#_Toc220878022)

[Contact matrices 7](#_Toc220878023)

[Force of infection 8](#_Toc220878024)

[Model calibration 9](#_Toc220878025)

[Intervention model 12](#_Toc220878026)

# RSV model structure

We developed an age-structured compartmental model to simulate the transmission dynamics of respiratory syncytial virus (RSV) in the population. The model stratified the population into 11 age groups: 0–2 months, 3–5 months, 6–11 months, 12–23 months, 2–4 years, 5–19 years, 20–59 years, 60–64 years, 65–69 years, 70–74 years and 75 years and above. Population distribution for these age groups was derived from 2018 Scottish demographic data[16]. For infants under one year, specific population counts for each month of age were not available in this dataset. Therefore, to estimate the population sizes for the 0–2 months, 3–5 months, and 6–11 months age groups, we assumed a uniform distribution of births across the calendar year. The total population of infants under one year was then allocated to these three subgroups proportionally to the duration (in months) of each respective interval. The epidemiological states for each variable are described in Table S1. The key parameters, including the average duration of the infectious period, the average duration of infection-acquired immunity, and the reduction of the infectious period for secondary, third and subsequent infections relative to primary infections, were determined based on previous literature. Details of parameters are described in Table S2.

**Table S1. Description of the epidemiological state variables of RSV model.**

| **State** | **Description** |
| --- | --- |
| *M* | Infants protected by passive immunity from maternal antibodies. |
| $S_{n}^{i}$ | Individuals in age group $i$ who are susceptible to their $n$th RSV infection. |
| $I_{n}^{i}$ | Individuals in age group $i$ who are infectious with their $n$th RSV infection , potentially with symptoms severe enough to require hospitalisation. |
| $R_{n}^{i}$ | Individuals in age group $i$ who have recovered from their $n$th RSV infection and have temporary immunity acquired through natural infection. |
| $V_{n}^{i}$ | Individuals in age group $i$ who are vaccinated before the $n$th infection and assumed to be fully protected from infection. |
| ${VS}_{n}^{i}$ | Vaccinated individuals in age group $i$who were partially protected by immunisation program, and susceptible to the $n$th RSV infection. |
| ${VI}_{n}^{i}$ | Vaccinated individuals in age group $i$ experiencing a breakthrough infection (their $n$th infection), who are infectious but have a milder course of disease not requiring hospitalisation. |
| $C^{i}$ | Cumulative incidence of all RSV infections in age group $i$. |
| $CV^{i}$ | Cumulative incidence of breakthrough RSV infections in age group $i$. |

**Table S2. Description of the epidemiological parameters in the RSV model.**

| **Parameters** | **Description** | **Values** | **Sources** |
| --- | --- | --- | --- |
| $\mu_{birth}$ | Daily live births. | 151 | [17] |
| $\mu_{death}$ | Daily death. | 151 | [17] |
| $1/\eta^{i}$ | Aging rate from age group $i$ to age group $i+1$. | 0–2 months: 90 days  3–5 months: 90 days  6–11 months: 180 days  12–23 months: 365 days  2–4 years: 1095 days  5–19 years: 5475 days  20–59 years: 14600 days  60–64 years: 1825 days  65–69 years: 1825 days  70–74 years: 1825 days  75 years and above: 0 day | – |
| $1/\gamma$ | Average duration of infectious period. | 7 days | [18] |
| $1/\omega_{m}$ | Average duration of immunity from maternal antibodies. | 28 days | [19] |
| $1/\omega_{inf}$ | Average duration of immunity from infection. | 300 days | [15] |
| $\delta^{i}$ | Age-specific susceptibility to RSV infection. | Based on scenarios:  0.4–0.6 for 5–59 years  0.2– 0.4 for 60 years and above | [15, 20] |
| $\sigma_{1}$ | Reduced recovery rate for secondary infection relative to primary infection. | 1.37 | [21, 22] |
| $\sigma_{2}$ | Reduced recovery rate for subsequent infections relative to secondary infection. | 1 |  |

The ODEs of the RSV transmission model for age group $i$ are as follows:

$$\frac{dM}{dt}=\mu-\omega_{m}$$

$$\frac{dS_{1}^{i}}{dt}=\omega_{m}-\lambda^{i}\left( t \right)\delta^{i}S_{1}^{i}-\eta^{i}S_{1}^{i}+\eta^{i-1}S_{1}^{i-1}$$

$$\frac{dI_{1}^{i}}{dt}=\lambda^{i}\left( t \right)\delta^{i}S_{1}^{i}-\gamma I_{1}^{i}-\eta^{i}I_{1}^{i}+\eta^{i-1}I_{1}^{i-1}$$

$$\frac{dR_{1}^{i}}{dt}=\gamma I_{1}^{i}-\omega_{inf}R_{1}^{i}-\eta^{i}R_{1}^{i}+\eta^{i-1}R_{1}^{i-1}$$

$$\frac{dS_{2}^{i}}{dt}=\omega_{inf}R_{1}^{i}-\lambda^{i}\left( t \right)\delta^{i}S_{2}^{i}-\eta^{i}S_{2}^{i}+\eta^{i-1}S_{2}^{i-1}$$

$$\frac{dI_{2}^{i}}{dt}=\lambda^{i}\left( t \right)\delta^{i}S_{2}^{i}-\gamma\sigma_{1}I_{2}^{i}-\eta^{i}I_{2}^{i}+\eta^{i-1}I_{2}^{i-1}$$

$$\frac{dR_{2}^{i}}{dt}=\gamma\sigma_{1}I_{2}^{i}-\omega_{inf}R_{2}^{i}-\eta^{i}R_{2}^{i}+\eta^{i-1}R_{2}^{i-1}$$

$$\frac{dS_{3}^{i}}{dt}=\omega_{inf}R_{2}^{i}+\omega_{inf}R_{3}^{i}-\lambda^{i}\left( t \right)\delta^{i}S_{3}^{i}-\eta^{i}S_{3}^{i}+\eta^{i-1}S_{3}^{i-1}$$

$$\frac{dI_{3}^{i}}{dt}=\lambda^{i}\left( t \right)\delta^{i}S_{3}^{i}-\gamma\sigma_{1}\sigma_{2}I_{3}^{i}-\eta^{i}I_{3}^{i}+\eta^{i-1}I_{3}^{i-1}$$

$$\frac{dR_{3}^{i}}{dt}=\gamma\sigma_{1}\sigma_{2}I_{3}^{i}-\omega_{inf}R_{3}^{i}-\eta^{i}R_{3}^{i}+\eta^{i-1}R_{3}^{i-1}$$

$$\frac{dC^{i}}{dt}=\lambda^{i}\left( t \right)\delta^{i}S_{1}^{i}+\lambda^{i}\left( t \right)\delta^{i}S_{2}^{i}+\lambda^{i}\left( t \right)\delta^{i}S_{3}^{i}$$

The differential equations included three components: transmission terms, demographics terms and ageing terms. Maternal immunity was incorporated exclusively in the first age group ($i=1$), providing temporary protection to newborns. The ageing process was modelled as a flow of individuals from age group $i$ to $i+1$ at an age-specific rate $\eta^{i}$. The demographics terms included a constant birth rate into the first age group ($i=1$) and age-specific mortality rates $\mu_{death}$ applied to age groups 60 years and above ($i>8$). This structure allows the model to maintain a stable population size where total births are balanced by total deaths across specfic age groups. The force of infection for age group $i$ at time $t$, denoted as $\lambda^{i}\left( t \right)$, varies by age and time to reflect seasonal RSV transmission patterns. A separate equation tracks the cumulative incidence of new infections ($C^{i}$) as a measure of the total infection burden. A proportion of infections, determined by the age-specific hospitalisation ratio, $Pr\left( H/I \right)$ resulted in hospitalisations. Relationships between epidemiological state variables are illustrated schematically in Figure S1.

1. **Model structure of the transmission model.**

Each panel represents RSV transmission within the age group $i$. In each age group, the population could experience multiple infections, and we do not distinguish infections over three times. Among the infected population, a proportion $Pr\left( H/I \right)$ required hospitalisation. Newborns entered the first age group through compartment *M*, which modelled maternal immunity. Ageing between groups was implemented through transitions from age group $i$ to $i+1$ at rate $\eta^{i}$. In age groups 60 years and above ($i>8$), mortality occurred at rate $\mu_{death}$ to maintain demographic equilibrium.

# Contact matrices

To estimate age-specific contact rates, we synthesized data from two UK social contact surveys: The POLYMOD study which provided a contact matrix for 5-year age groups (ranging from 0–4 years to 75 years and above) [23], and a UK contact survey that reported contact patterns between infants under 1 year and other age groups[24]. We converted the reported daily contact numbers into rates by normalizing them against the corresponding age-specific population data from the years when the surveys were conducted. We then calculated age-specific contact rates with adjustments for changes in population age distribution between the original contact surveys and our study period[25].

The contact rates per capita $c^{I,J}$ between age groups $I$ and $J$ were calculated as:

$$c^{I,J}=\frac{P^{I,J}N^{J}+P^{J,I}N^{I}}{2*N^{I}*N^{J}}$$

Where $P^{I,J}$ represents the average daily number of contacts reported by contact survey in the age group $I$ with those in age group $J$, and $N^{I}$ and $N^{J}$ denotes the population sizes of the relative age groups in our study. This transformation ensured symmetry of contact rates and standardised them to our study population.

We then integrated these two contact rate datasets and restructured them to match the age structure of our model: 0–2 months, 3–5 months, 6–11 months, 12–23 months, 2–4 years, 5–19 years, 20–59 years, 60–64 years, 65–69 years, 70–74 years, and 75 years and above. The contact rate between an infant interacting with one another was estimated using the contact matrix for children under 5 years, and the contact matrix between infants under 1 year and children under 5 years; We assumed that the contact rate between children aged 1–4 years interacting with children under 5 years was equivalent to the contact rate for a child under 5 years interacting with one another. Contact rates were assumed to be equivalent between narrower age bands among infants, and between narrower age bands among children aged 1–4 years. For adults and older adults, we calculated the contact rates for 5–19 years and 20–59 years by aggregating 5-year age groups, and assumed a constant contact rate for all individuals aged 70 years and above. This aggregation involved three steps.

First, we calculated the total contacts $T^{I,J}$ between the original age groups reported by the two contact surveys:

$$T^{I,J}=c^{I,J}*N^{I}*N^{J}$$

Where $N^{I}$ and $N^{J}$ are the population sizes of the original age groups.

Second, we calculate total contacts across aggregated groups. For aggregated age groups $i$ and $j$, total contacts were calculated as:

$$T^{i,j}=\sum_{I}^{i} \sum_{J}^{j} T^{I,J}$$

Finally, we calculated the contact rates per capita for the aggregated age groups and ensured that the final contact matrix was symmetric as follow:

$$C^{i,j}=\frac{T^{i,j}}{N_{i}*N_{j}}$$

Where $N_{i}$ and $N^{J}$ are the population sizes of the aggregated age groups.

# Force of infection

The force of infection in our model integrated two components: a time-varying transmission rate $\beta\left( t \right)$ and a contact matrix $C^{i,j}$. The transmission rate $\beta\left( t \right)$ comprised two terms: a baseline transmission rate $\beta_{\text{base}}$, representing the minimum annual transmission, and a seasonal term that captures temporal variations in transmission intensity.

The seasonal component followed a normal distribution, reflecting the single-peak annual RSV transmission pattern with low baseline transmission outside the season in a year. This distribution was parameterised with three values: the peak timing $\phi$ (fixed in week 47), the mean seasonal transmission rate $\beta_{\text{seasonal}}$ and the temporal spread $\psi$, corresponding to the standard deviation of the seasonal curve. The contact matrix $C^{i,j}$ captured age-specific social mixing patterns, quantifying the average per capita contact rate between individuals in age group $i$ and those in age group $j$. The force of infection for age group $i$ at time $t$ was calculated as:

$$\lambda^{i}\left( t \right)=\left( \beta_{\text{base}}+\beta_{\text{seasonal}}\cdot\frac{1}{\psi\sqrt{2\pi}}\exp\left( -\frac{1}{2}\left( \frac{t-\phi}{\psi} \right)^{2} \right) \right)\sum_{j=1}^{11} C^{i,j}\left( I_{0}^{j}+I_{1}^{j}+I_{2}^{j} \right)$$

# Model calibration

We used a multi-step calibration framework to estimate model parameters, incorporating a preliminary characterisation of seasonality followed by a two-stage Bayesian inference strategy to determine age-specific susceptibility and transmission dynamics.

Prior to the main calibration, we estimated the temporal spread parameter $\psi$ to characterise the seasonal wave profile of RSV. Observational data from March 2017 to March 2020 were aggregated into 52 weekly intervals. We characterized RSV seasonal epidemic patterns using the Annual Average Percentage (AAP) method, which quantifies relative viral activity intensity through the equation:

$$AAP=\sum_{1}^{52} \frac{n_{i}}{\sum_{1}^{52} n_{i}}\times100\%$$

Where $n_{i}$ represents the weekly case count in week $i$. Epidemic onset and offset were defined as periods where AAP values exceeded a 75% threshold. We generated a target seasonal curve using a normal distribution sequence over 52 weeks and iteratively optimised the standard deviation ($\psi$) using simulated annealing to minimise the deviation between simulated and empirical AAP values, thereby aligning the model with observed seasonal trends.

We modelled age-specific susceptibility for three age groups, i.e., individuals under 5 years, 5–59 years, and 60 years and above. Children under 5 years served as the reference group with a fixed susceptibility coefficient of 1.0, reflecting their high baseline risk. Susceptibility coefficients for the 5–59 and ≥60 years groups were estimated using a two-stage strategy that integrated a profile likelihood approach with Markov Chain Monte Carlo (MCMC) sampling.

In the first stage, we performed model calibration against weekly RSV-positive case data (2017/18–2019/20) to optimise transmission and reporting parameters across a predefined grid of susceptibility coefficients. Based on age-specific risk profiles from cohort studies, the grid was designed to include 176 value pairs for the two susceptibility coefficients, spanning 0.3–0.6 for the 5–59 years group and 0.2–0.4 for the ≥60 years group, both in increments of 0.02.

For each fixed pair of susceptibility coefficients, we estimated 14 parameters (three transmission parameters and 11 age-specific reporting rates) using MCMC. Simulations were initialised on January 1, 1999, with ten infectious individuals seeded per age group, running until equilibrium was reached prior to data fitting. We adopted a joint likelihood function combining a negative binomial distribution for weekly case counts and a Dirichlet-multinomial distribution for the age stratification of cases (split before and after December to capture temporal shifts in age distribution). The joint likelihood function was calculated as:

$$\mathcal{L=}\sum_{i=1}^{11} \sum_{t} \log\left( f_{NegBinom}\left( y_{i,t};\mu_{i,t},r \right) \right)+\sum\log\left( f_{\text{DirMult}}\left( \mathbf{y}_{\mathbf{k}};N_{k},\boldsymbol{\mu}_{\boldsymbol{k}} \right) \right)$$

Where $y_{i,t}$ is the observed weekly RSV-positive test results from all laboratories in Scotland in age group $i$ at week $t$; $\mu_{i,t}$ is the model-predicted cases in age group $i$ at week $t$; $r$ is the dispersion parameter of the negative binomial distribution; $\mathbf{y}_{\mathbf{k}}$ is the observed age distribution of cases in time period $k$; $N_{k}$ is the total cases in time period $k$, and $\boldsymbol{\mu}_{\boldsymbol{k}}$ is the model-predicted age distribution for the same period. We used non-informative priors for all parameters. The MCMC process involved 10,000 burn-in iterations followed by 20,000 sampling iterations, and sampled every tenth iteration to reduce autocorrelation.

In the second stage, we identified the optimal susceptibility configuration by comparing model predictions with observed RSV infection rate data. For each susceptibility pair defined in Stage 1, we resampled 1,000 parameter sets from the corresponding posterior distribution and simulated infections. The goodness-of-fit was assessed using a Poisson likelihood function:

$$\mathcal{L}=x\log\left( \lambda\right)-\lambda-\log\left( x! \right)$$

where $x$ represents the observed number of infected individuals (calculated from published infection rates and population data), and $\lambda$ is the model-predicted number of cases. We selected the age-specific susceptibility parameter combination with the maximum likelihood as the optimal set. The 95% confidence intervals (CIs) of the susceptibility coefficients were estimated based on likelihood ratios.

# Intervention model

To evaluate the impact of immunisation programs, we simulated multiple intervention scenarios. These included variations in four parameters: immunisation coverage ($P_{\text{immu}}$), vaccine efficacy against infection ($E_{\text{inf}}$) and hospitalization ($E_{\text{hosp}}$), and eligible age groups for immunisation programs. The parameter definitions and values are provided in Table S3.

**Table S3. Description of the immunisation-associated parameters in the RSV model.**

| **Parameters** | **Description** | **Values** | **Sources** |
| --- | --- | --- | --- |
| $1/\omega_{\text{immu}}$ | Duration of the immunity from immunisation program. | 180 days | [10, 35, 36] |
| $E_{\text{inf}}$ | Efficacy of prevent infection. | Based on scenario: 60%, 70%, 80% | [10, 11, 35–38] |
| $E_{\text{hosp}}$ | Efficacy of prevent Hospitalisation. | Based on scenario: 70%, 80%, 90% | [10, 11, 35–38] |
| $P_{\text{immu}}$ | Coverage of immunisation plan. | Based on scenario: 60%, 80% |  |

The model assumed immunisation program initiation on 1^st^ September, preceding the typical RSV seasonal peak. For simplicity, we modelled immediate program rollout with instantaneous protection, disregarding logistical delays in vaccine delivery. Among the eligible population receiving the intervention (coverage $P_{\text{immu}}$), a proportion $P_{immu}E_{inf}S_{n}^{i}$ transitioned to the fully protected status ($V_{n}^{i}$), while $P_{immu}\left( E_{hosp}-E_{inf} \right)S_{n}^{i}$ acquired partial protection ($VS_{n}^{i}$), reducing hospitalisation risk but remaining susceptible to infection. The remaining $\left( 1-P_{immu} \right)S_{n}^{i}$ retained baseline susceptibility. Transitions between these epidemiological states are illustrated in Figure S2.

The ODEs of the RSV intervention model for the age group $i$ are as follows:

$$\frac{dM}{dt}=\mu-\omega_{m}$$

$$\frac{dS_{1}^{i}}{dt}=\omega_{m}-\lambda^{i}\left( t \right)\delta^{i}S_{1}^{i}-P_{immu}E_{inf}S_{1}^{i}-P_{immu}\left( E_{hosp}-E_{inf} \right)S_{1}^{i}+\omega_{immu}V_{1}^{i}+\omega_{immu}S_{V1}^{i}-\eta^{i}S_{1}^{i}+\eta^{i-1}S_{1}^{i-1}$$

$$\frac{dV_{1}^{i}}{dt}=P_{immu}E_{inf}S_{1}^{i}-\omega_{immu}V_{1}^{i}-\eta^{i}V_{1}^{i}+\eta^{i-1}V_{1}^{i-1}$$

$$\frac{dS_{V1}^{i}}{dt}=P_{immu}\left( E_{hosp}-E_{inf} \right)S_{1}^{i}-\lambda^{i}\left( t \right)\delta^{i}S_{V1}^{i}-\omega_{immu}S_{V1}^{i}-\eta^{i}S_{V1}^{i}+\eta^{i-1}S_{V1}^{i-1}$$

$$\frac{dI_{1}^{i}}{dt}=\lambda^{i}\left( t \right)\delta^{i}S_{1}^{i}-\gamma I_{1}^{i}-\eta^{i}I_{1}^{i}+\eta^{i-1}I_{1}^{i-1}$$

$$\frac{dI_{V1}^{i}}{dt}=\lambda^{i}\left( t \right)\delta^{i}S_{V1}^{i}-\gamma I_{V1}^{i}-\eta^{i}I_{V1}^{i}+\eta^{i-1}I_{V1}^{i-1}$$

$$\frac{dR_{1}^{i}}{dt}=\gamma I_{1}^{i}+\gamma I_{V1}^{i}-\omega_{inf}R_{1}^{i}-\eta^{i}R_{1}^{i}+\eta^{i-1}R_{1}^{i-1}$$

$$\frac{dS_{2}^{i}}{dt}=\omega_{inf}R_{1}^{i}-\lambda^{i}\left( t \right)\delta^{i}S_{2}^{i}-P_{immu}E_{inf}S_{2}^{i}-P_{immu}\left( E_{hosp}-E_{inf} \right)S_{2}^{i}+\omega_{immu}S_{V2}^{i}+\omega_{immu}V_{2}^{i}-\eta^{i}S_{2}^{i}+\eta^{i-1}S_{2}^{i-1}$$

$$\frac{dV_{2}^{i}}{dt}=P_{immu}E_{inf}S_{2}^{i}-\omega_{immu}V_{2}^{i}-\eta^{i}V_{2}^{i}+\eta^{i-1}V_{2}^{i-1}$$

$$\frac{dS_{V2}^{i}}{dt}=P_{immu}\left( E_{hosp}-E_{inf} \right)S_{2}^{i}-\lambda^{i}\left( t \right)\delta^{i}S_{V2}^{i}-\omega_{immu}S_{V2}^{i}-\eta^{i}S_{V2}^{i}+\eta^{i-1}S_{V2}^{i-1}$$

$$\frac{dI_{2}^{i}}{dt}=\lambda^{i}\left( t \right)\delta^{i}S_{2}^{i}-\gamma\sigma_{1}I_{2}^{i}-\eta^{i}I_{2}^{i}+\eta^{i-1}I_{2}^{i-1}$$

$$\frac{dI_{V2}^{i}}{dt}=\lambda^{i}\left( t \right)\delta^{i}S_{V2}^{i}-\gamma\sigma_{1}I_{V2}^{i}-\eta^{i}I_{V2}^{i}+\eta^{i-1}I_{V2}^{i-1}$$

$$\frac{dR_{2}^{i}}{dt}=\gamma\sigma_{1}I_{2}^{i}+\gamma\sigma_{1}I_{V2}^{i}-\omega_{inf}R_{2}^{i}-\eta^{i}R_{2}^{i}+\eta^{i-1}R_{2}^{i-1}$$

$$\frac{dS_{3}^{i}}{dt}=\omega_{inf}R_{2}^{i}+\omega_{inf}R_{3}^{i}-\lambda^{i}\left( t \right)\delta^{i}S_{3}^{i}-P_{immu}E_{inf}S_{3}^{i}-P_{immu}\left( E_{hosp}-E_{inf} \right)S_{3}^{i}+\omega_{immu}S_{V3}^{i}+\omega_{immu}V_{3}^{i}-\eta^{i}S_{3}^{i}+\eta^{i-1}S_{3}^{i-1}$$

$$\frac{dV_{3}^{i}}{dt}=P_{immu}E_{inf}S_{3}^{i}-\omega_{immu}V_{3}^{i}-\eta^{i}V_{3}^{i}+\eta^{i-1}V_{3}^{i-1}$$

$$\frac{dS_{V3}^{i}}{dt}=P_{immu}\left( E_{hosp}-E_{inf} \right)S_{3}^{i}-\lambda^{i}\left( t \right)\delta^{i}S_{V3}^{i}-\omega_{immu}S_{V3}^{i}-\eta^{i}S_{V3}^{i}+\eta^{i-1}S_{V3}^{i-1}$$

$$\frac{dI_{3}^{i}}{dt}=\lambda^{i}\left( t \right)\delta^{i}S_{3}^{i}-\gamma\sigma_{1}\sigma_{2}I_{3}^{i}-\eta^{i}I_{3}^{i}+\eta^{i-1}I_{3}^{i-1}$$

$$\frac{dI_{V3}^{i}}{dt}=\lambda^{i}\left( t \right)\delta^{i}S_{V3}^{i}-\gamma\sigma_{1}\sigma_{2}I_{V3}^{i}-\eta^{i}I_{V3}^{i}+\eta^{i-1}I_{V3}^{i-1}$$

$$\frac{dR_{3}^{i}}{dt}=\gamma\sigma_{1}\sigma_{2}I_{3}^{i}+\gamma\sigma_{1}\sigma_{2}I_{V3}^{i}-\omega_{inf}R_{3}^{i}-\eta^{i}R_{3}^{i}+\eta^{i-1}R_{3}^{i-1}$$

$$\frac{dC^{i}}{dt}=\lambda^{i}\left( t \right)\delta^{i}S_{1}^{i}+\lambda^{i}\left( t \right)\delta^{i}S_{2}^{i}+\lambda^{i}\left( t \right)\delta^{i}S_{3}^{i}$$

$$\frac{dCV^{i}}{dt}=\lambda^{i}\left( t \right)\delta^{i}S_{V1}^{i}+\lambda^{i}\left( t \right)\delta^{i}S_{V2}^{i}+\lambda^{i}\left( t \right)\delta^{i}S_{V3}^{i}$$


1. **Model structure of the transmission model with immunisation program.**

Each panel represents RSV transmission within the age group $i$, stratified by infection history (1st to 3rd infections). The population in each age group could experience multiple infections. Following the $n^{th}$ infection, the population lost immunity at rate $\omega_{inf}$ and transitioned to susceptibility for the $\left( n+1 \right)^{th}$ infection. Among the infected population, a proportion $Pr\left( H/I \right)$ required hospitalisation. Newborns entered the first age group through compartment *M*, which modelled maternal immunity. Ageing between groups was implemented through transitions from age group $i$ to $i+1$ at rate $\eta^{i}$. In age groups 60 years and above ($i>8$), mortality occurred at rate $\mu_{death}$ to maintain demographic equilibrium.
