# Supplementary material for: The influence of age-dependent susceptibility on RSV transmission dynamics and immunisation population-level impact
Source: BMC Med. 2026 Mar 11;24:254. doi: 10.1186/s12916-026-04776-1 (PMC13088621; doi:10.1186/s12916-026-04776-1)
Supplement: Supplementary file 2 — Additional file 2: Figure S1. Trace plots of the parameters for the base scenario. Figure S2. Trace plots of the parameters for the best-fitting model. Figure S3. Population-wide averted infections and hospitalisations under the base model. Figure S4. Population-wide averted infections and hospitalisations under best fitting model. Figure S5. Population-wide averted infections and hospitalisations under the base model with infection-hospitalisation ratios derived from best-fitting model. Figure S6. Population-wide proportion of averted infections and hospitalisations in all ages combined under the base model. Figure S7. Population-wide proportion of averted infections and hospitalisations in all age combined under best-fitting model. Figure S8. Population-wide proportion of averted infections and hospitalisations in all age combined under the base model with infection-hospitalisation ratios derived from best-fitting model. Figure S9. The number of people need to be immunised to prevent one RSV hospitalisation under the base model. Figure S10. The number of people need to be immunised to prevent one RSV hospitalisation under best-fitting model. Figure S11. The number of people needed to be immunised to prevent one RSV hospitalisation under the base model with infection-hospitalisation ratios derived from best-fitting model. Table S1. Hospitalisation rate from previous research. Table S2. Estimated infection rate for infant and log-likelihood under different combinations of age-specific parameters. Table S3. Parameter estimates from different models. [file 12916_2026_4776_MOESM2_ESM.docx]

**Additional file 2 for The influence of age-dependent susceptibility on RSV transmission dynamics and immunisation population-level impact**

**Results**

Chenkai Zhao, Yuhe Zhang, Richard Osei-Yeboah, Xiao Li, You Li, Xin Wang, Harish Nair

Contents

[Figure S1. Trace plots of the parameters for the base scenario. 3](#_Toc220767697)

[Figure S2. Trace plots of the parameters for the best-fitting model. 4](#_Toc220767698)

[Figure S3. Population-wide averted infections and hospitalisations under the base model. 5](#_Toc220767699)

[Figure S4. Population-wide averted infections and hospitalisations under best fitting model. 6](#_Toc220767700)

[Figure S5. Population-wide averted infections and hospitalisations under the base model with infection-hospitalisation ratios drived from best-fitting model. 7](#_Toc220767701)

[Figure S6. Population-wide proportion of averted infections and hospitalisations in all age combined under the base model. 8](#_Toc220767702)

[Figure S7. Population-wide proportion of averted infections and hospitalisations in all age combined under best-fitting model. 9](#_Toc220767703)

[Figure S8. Population-wide proportion of averted infections and hospitalisations in all age combined under the base model with infection-hospitalisation ratios drived from best-fitting model . 10](#_Toc220767704)

[Figure S9. The number of people need to be immunised to prevent one RSV hospitalisation under the base model. 11](#_Toc220767705)

[Figure S10. The number of people need to be immunised to prevent one RSV hospitalisation under best-fitting model. 12](#_Toc220767706)

[Figure S11. The number of people need to be immunised to prevent one RSV hospitalisation under the base model with infection-hospitalisation ratios drived from best-fitting model. 13](#_Toc220767707)

[Table S1. Hospitalisation rate from previously research. 14](#_Toc220767708)

[Table S2. Estimated infection rate for infant and log-likelihood under different combination of age-specific parameters. 15](#_Toc220767709)

[Table S3. Parameter estimates from different models. 23](#_Toc220767710)

**
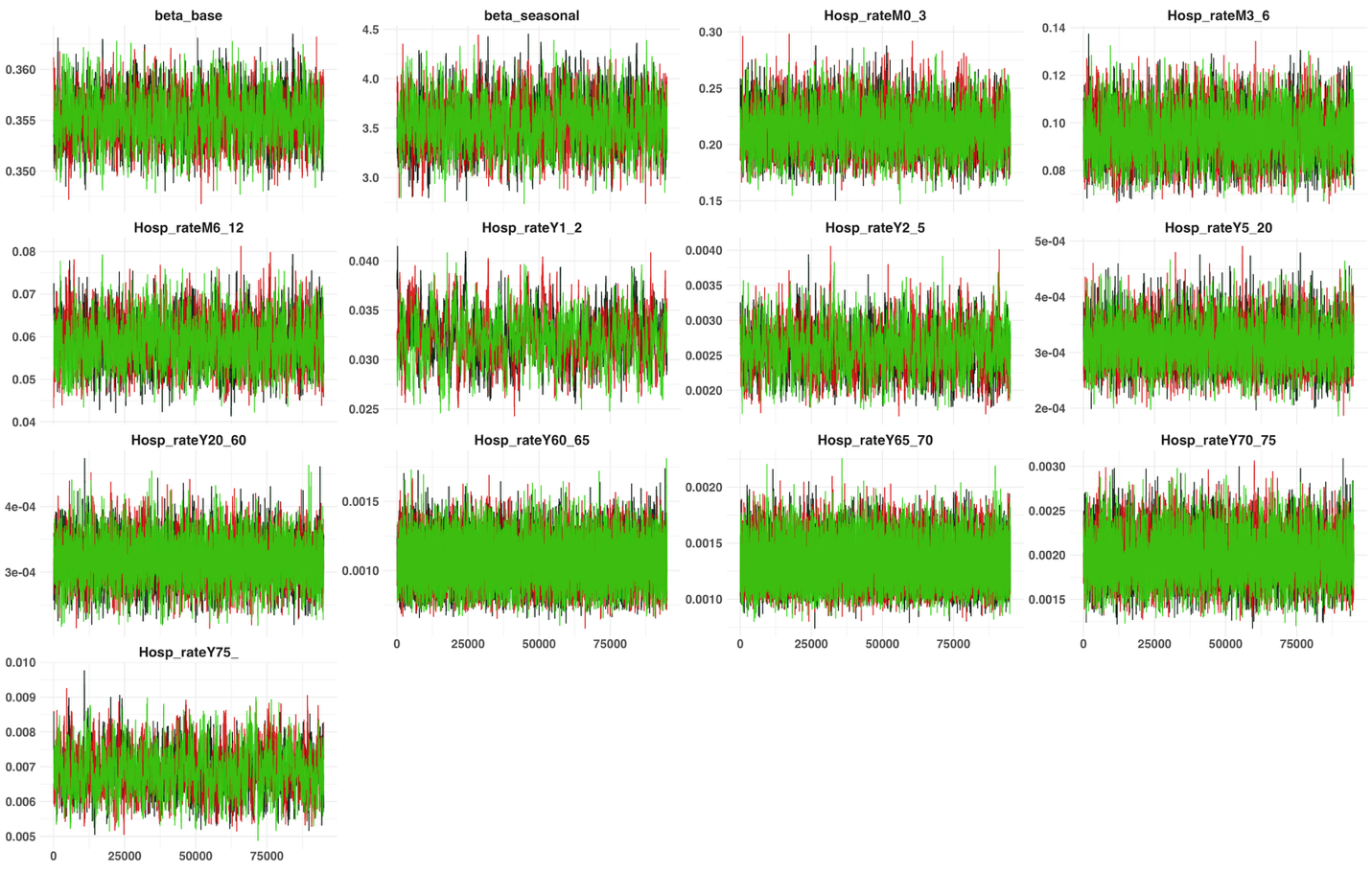
**

# Figure S1. Trace plots of the parameters for the base scenario.

**
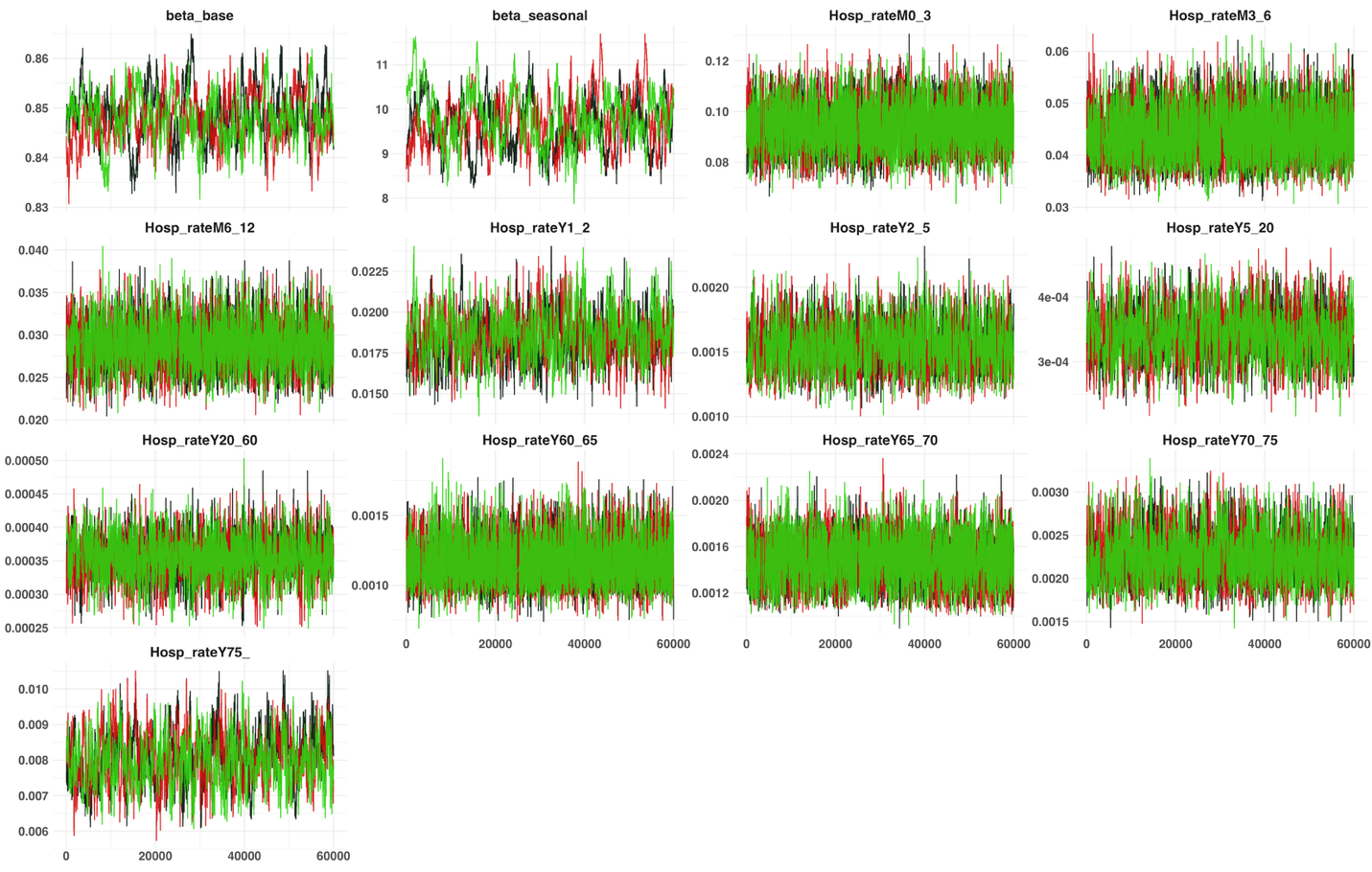
**

# Figure S2. Trace plots of the parameters for the best-fitting model.

#
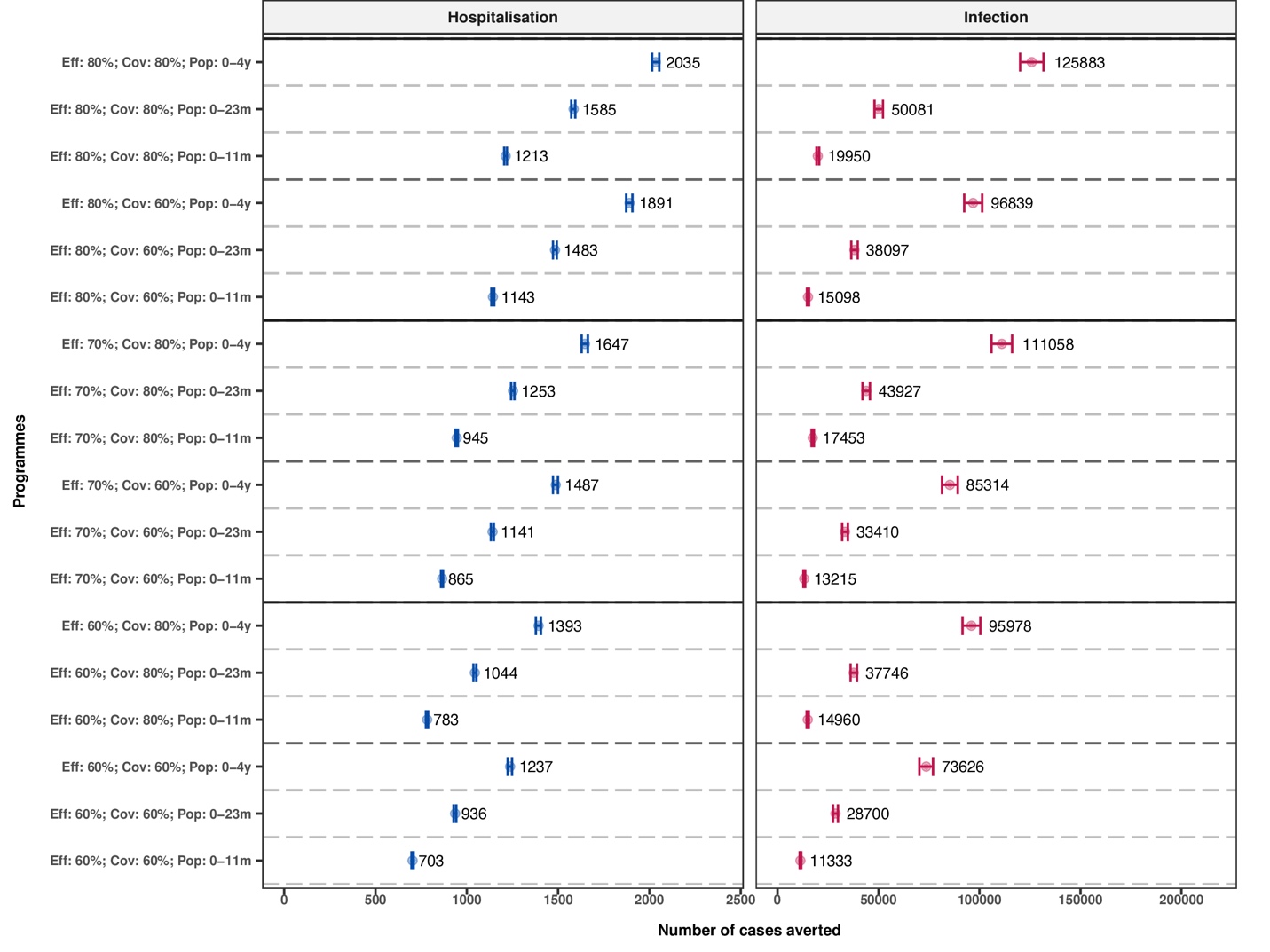
Figure S3. Population-wide averted infections and hospitalisations under the base model.

The figure illustrates the total number of averted hospitalisations and infections across the entire population under varying immunisation parameters. Estimates incorporate both direct protection in the eligible population (Pop) and indirect immunity effects in unvaccinated age groups. Results are derived from the homogeneous susceptibility baseline scenario, where susceptibility coefficients were set to 1.0 for all age groups (<5, 5–59, and ≥60 years). Eff=efficacy of immunisation programme. Cov=coverage of immunisation programme.

#
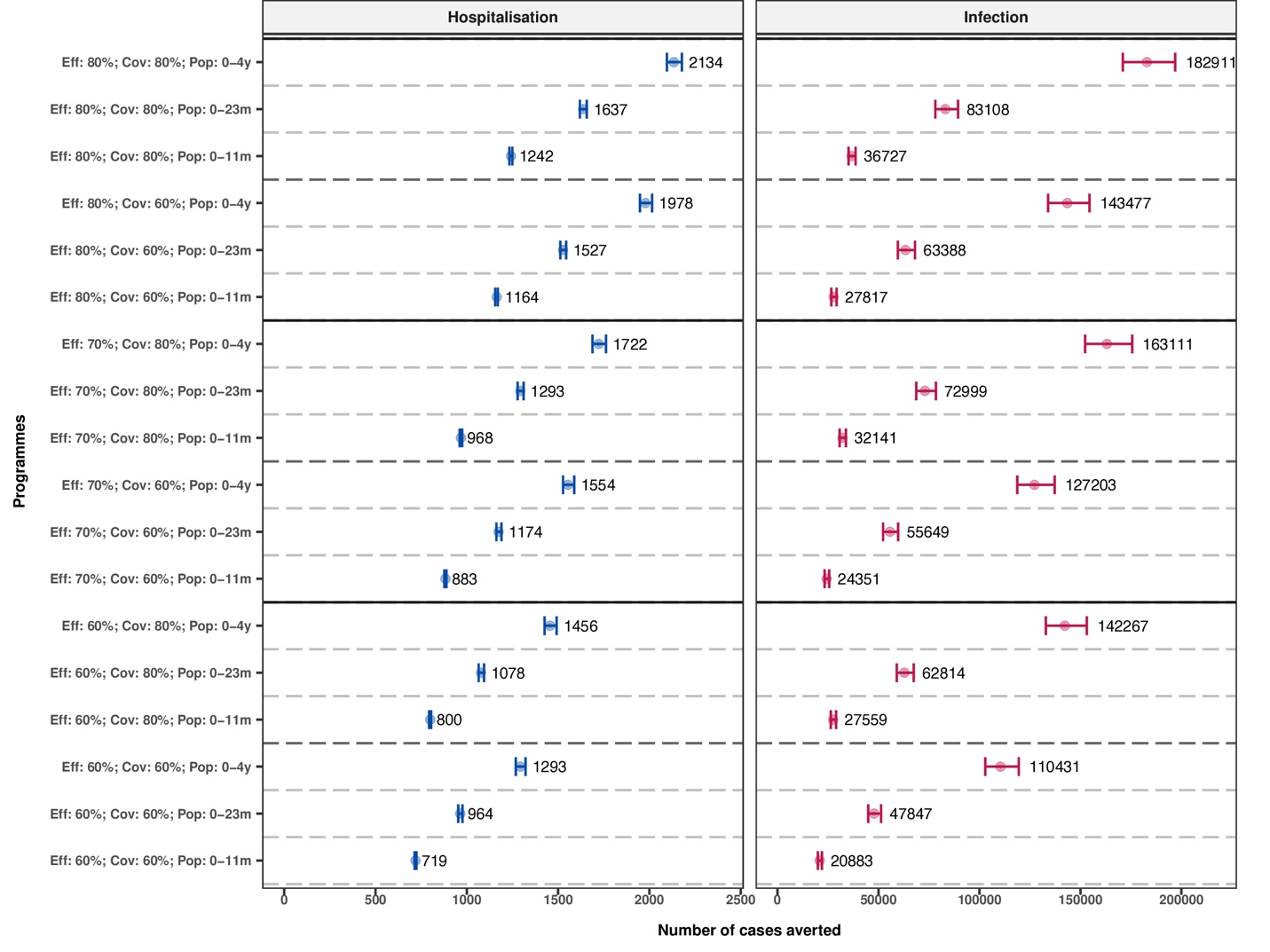
Figure S4. Population-wide averted infections and hospitalisations under best fitting model.

The figure illustrates the total number of averted hospitalisations and infections across the entire population under varying immunisation parameters. Estimates incorporate both direct protection in the eligible population (Pop) and indirect immunity effects in unvaccinated age groups. Results are derived from the homogeneous susceptibility baseline scenario. The susceptibility coefficient for 0–4 years old, 5–59 years old and 60 years and above is 1.0, 0.38 and 0.38. Eff=efficacy of immunisation programme. Cov=coverage of immunisation programme.

#
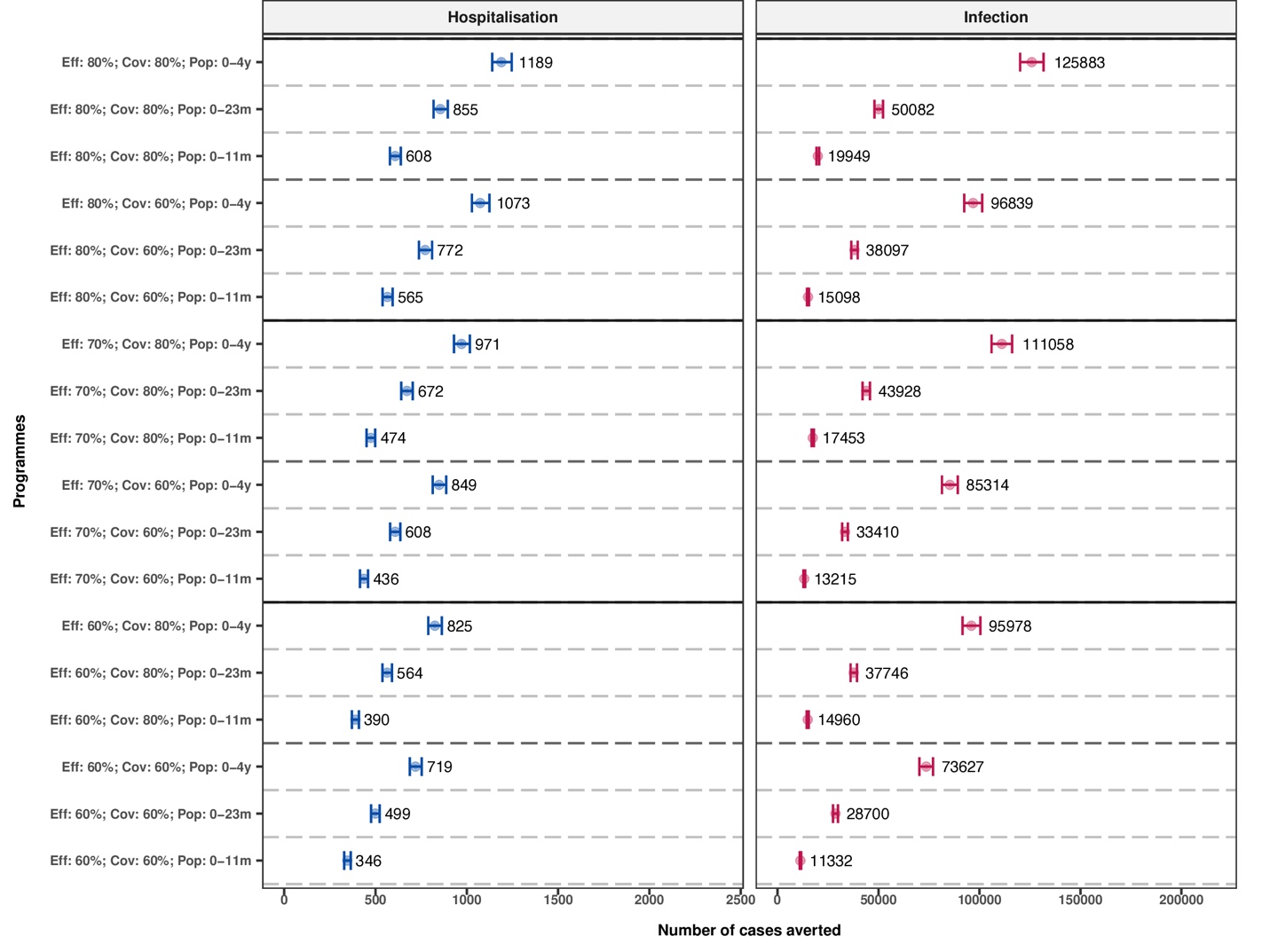
Figure S5. Population-wide averted infections and hospitalisations under the base model with infection-hospitalisation ratios drived from best-fitting model.

The figure illustrates the total number of averted hospitalisations and infections across the entire population under varying immunisation parameters. Estimates incorporate both direct protection in the eligible population (Pop) and indirect immunity effects in unvaccinated age groups. Results are derived from the homogeneous susceptibility baseline scenario, where susceptibility coefficients were set to 1.0 for all age groups (<5, 5–59, and ≥60 years). Eff=efficacy of immunisation programme. Cov=coverage of immunisation programme.

#
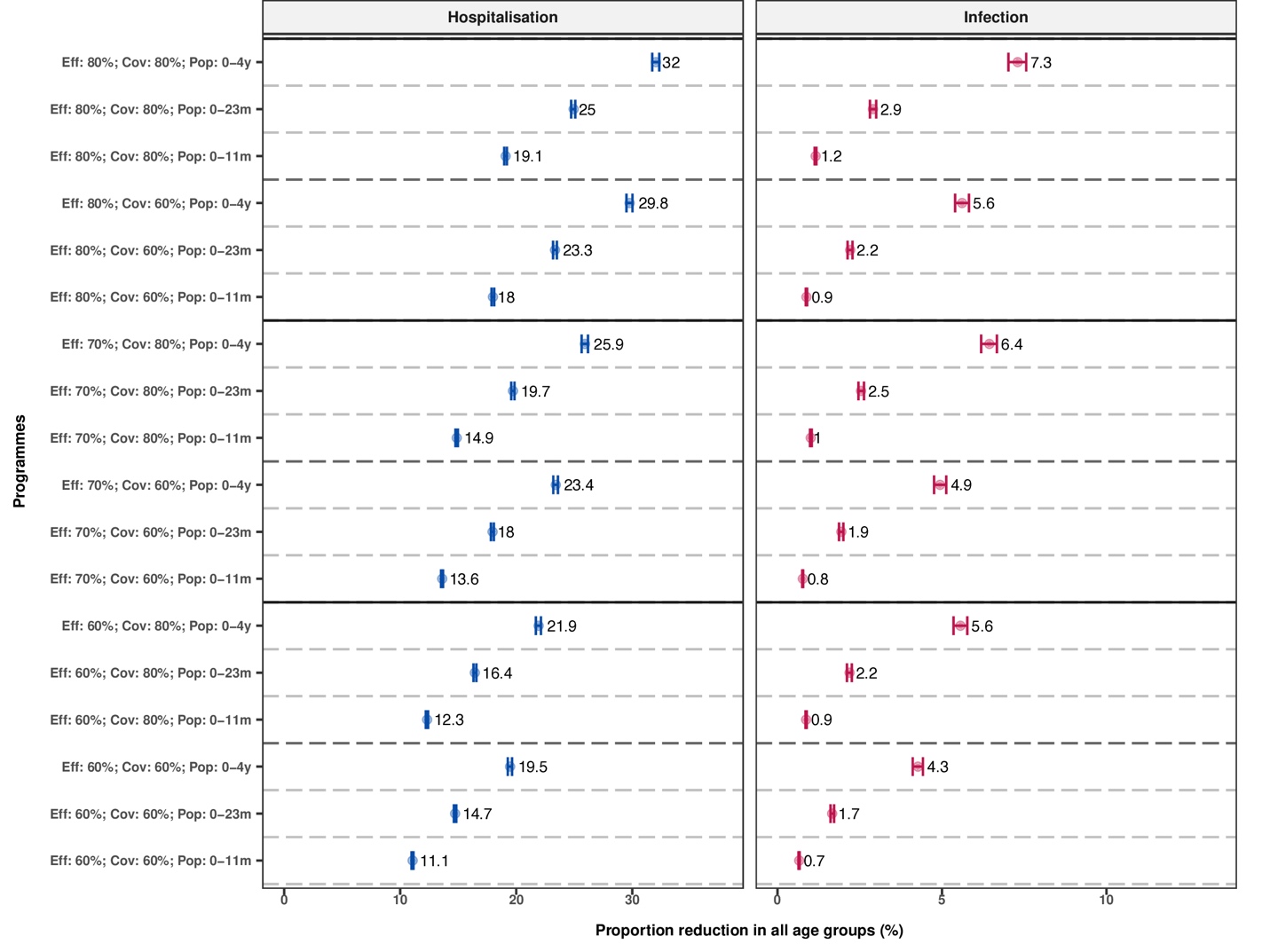
Figure S6. Population-wide proportion of averted infections and hospitalisations in all age combined under the base model.

The figure illustrates the total number of averted proportion of hospitalisations and infections across the entire population under varying immunisation parameters. Estimates incorporate both direct protection in the eligible population (Pop) and indirect immunity effects in unvaccinated age groups. Results are derived from the homogeneous susceptibility baseline scenario, where susceptibility coefficients were set to 1.0 for all age groups (<5, 5–59, and ≥60 years). Eff=efficacy of immunisation programme. Cov=coverage of immunisation programme.


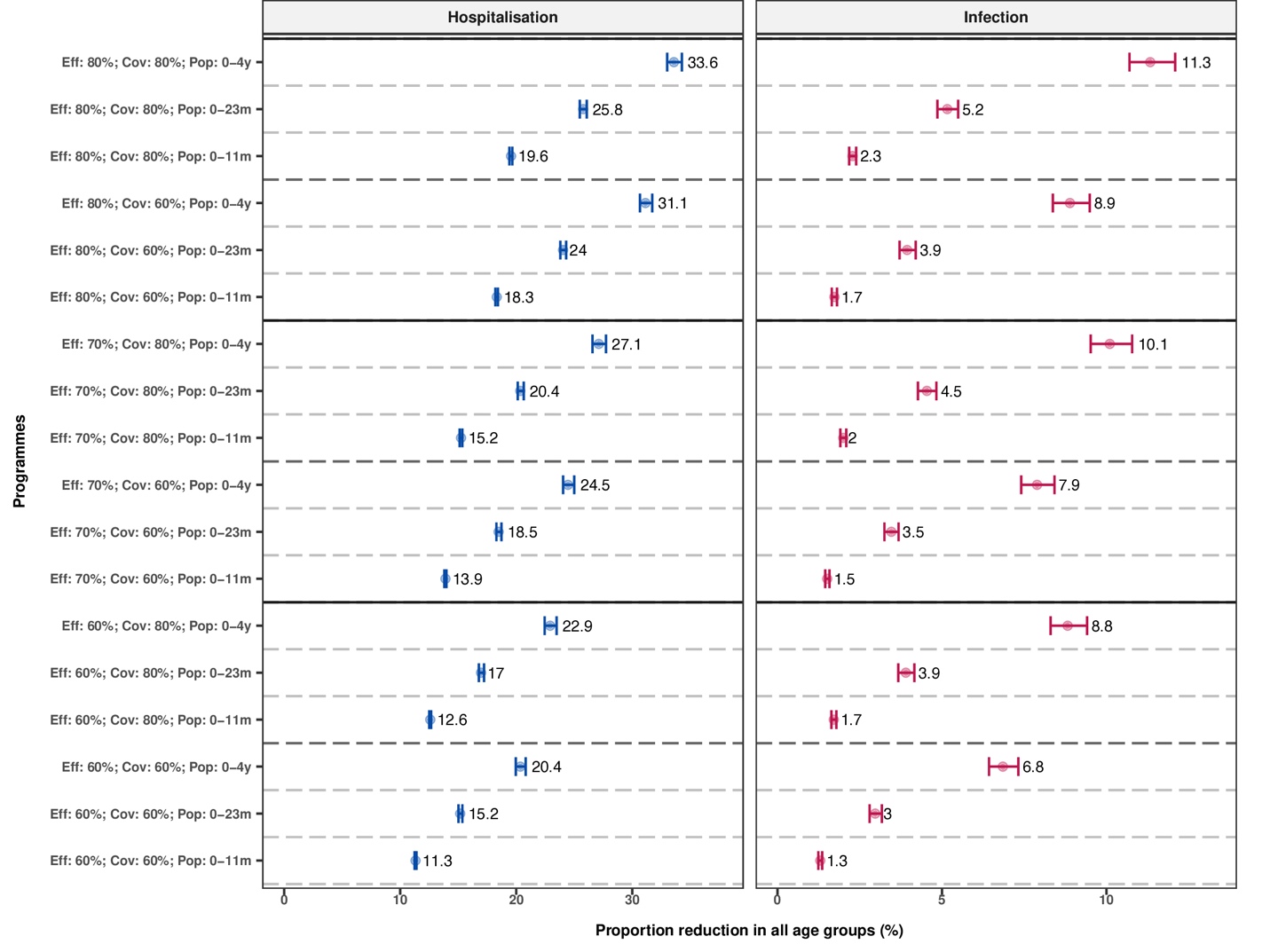


# Figure S7. Population-wide proportion of averted infections and hospitalisations in all age combined under best-fitting model.

The figure illustrates the total number of averted proportion of hospitalisations and infections across the entire population under varying immunisation parameters. Estimates incorporate both direct protection in the eligible population (Pop) and indirect immunity effects in unvaccinated age groups. Results are derived from the homogeneous susceptibility baseline scenario. The susceptibility coefficient for 0–4 years old, 5–59 years old and 60 years and above is 1.0, 0.38 and 0.38. Eff=efficacy of immunisation programme. Cov=coverage of immunisation programme.


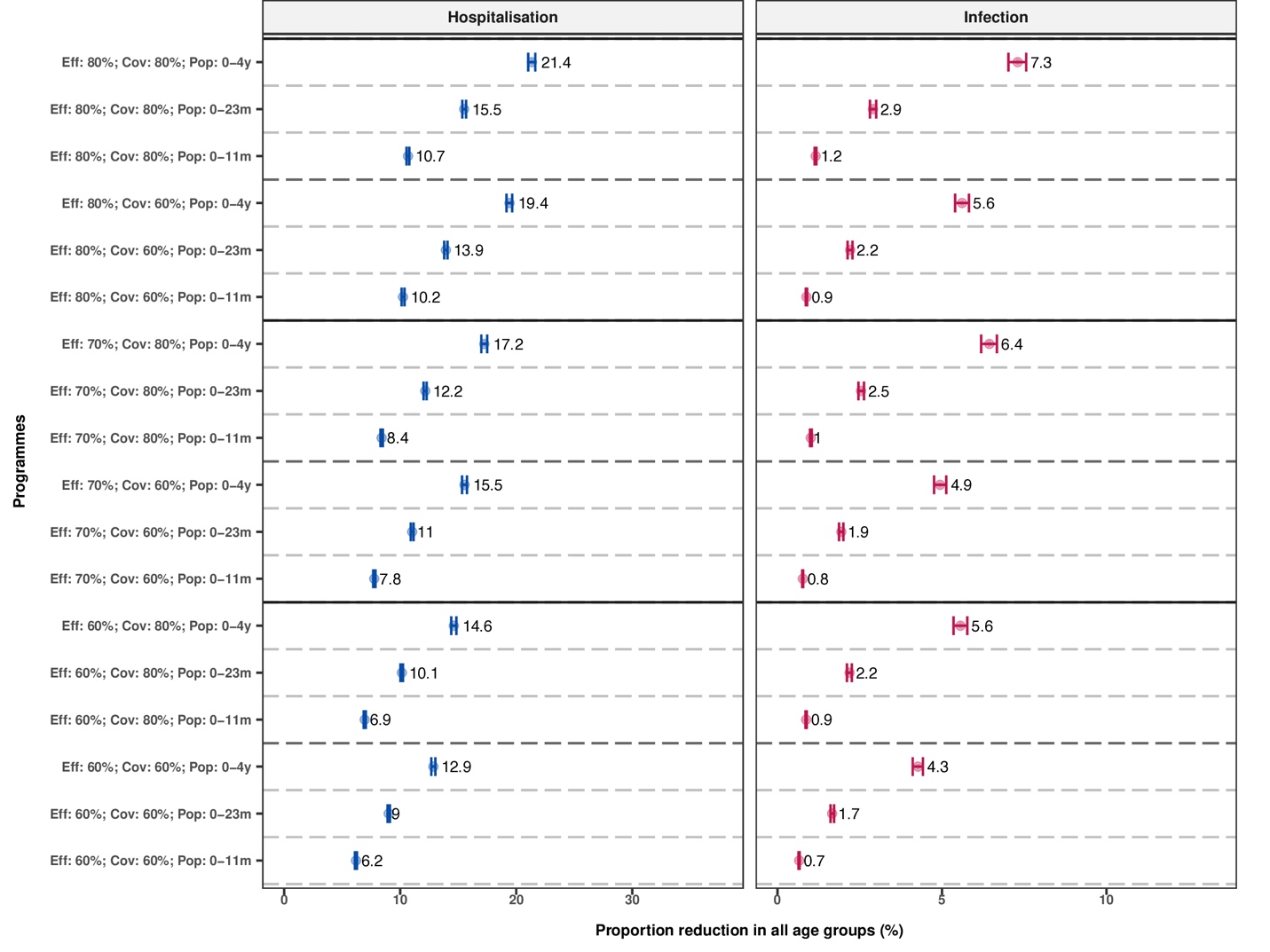


# Figure S8. Population-wide proportion of averted infections and hospitalisations in all age combined under the base model with infection-hospitalisation ratios drived from best-fitting model .

The figure illustrates the total number of averted proportion of hospitalisations and infections across the entire population under varying immunisation parameters. Estimates incorporate both direct protection in the eligible population (Pop) and indirect immunity effects in unvaccinated age groups. Results are derived from the homogeneous susceptibility baseline scenario, where susceptibility coefficients were set to 1.0 for all age groups (<5, 5–59, and ≥60 years). Eff=efficacy of immunisation programme. Cov=coverage of immunisation programme.


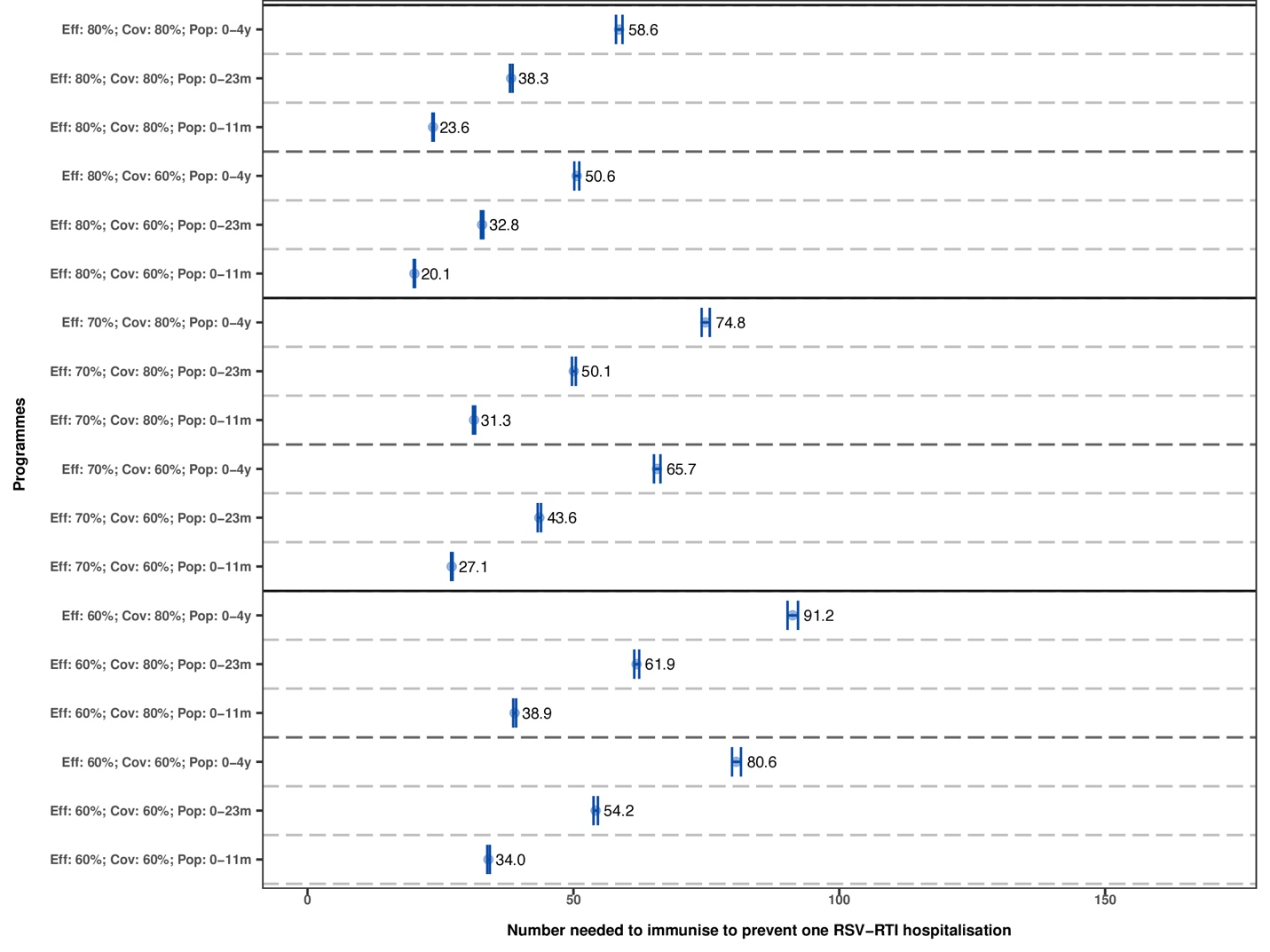


# Figure S9. The number of people need to be immunised to prevent one RSV hospitalisation under the base model.

This figure illustrates the number of people need to be vaccinated to avert one hospitalisation case based on varying immunisation parameters. Abbreviations: Eff, efficacy of immunisation programme; Cov, coverage of immunisation programme; Pop, eligible population. The susceptibility coefficient for 0–4 years old, 5–59 years old and 60 years and above is 1.0, 1.0 and 1.0.


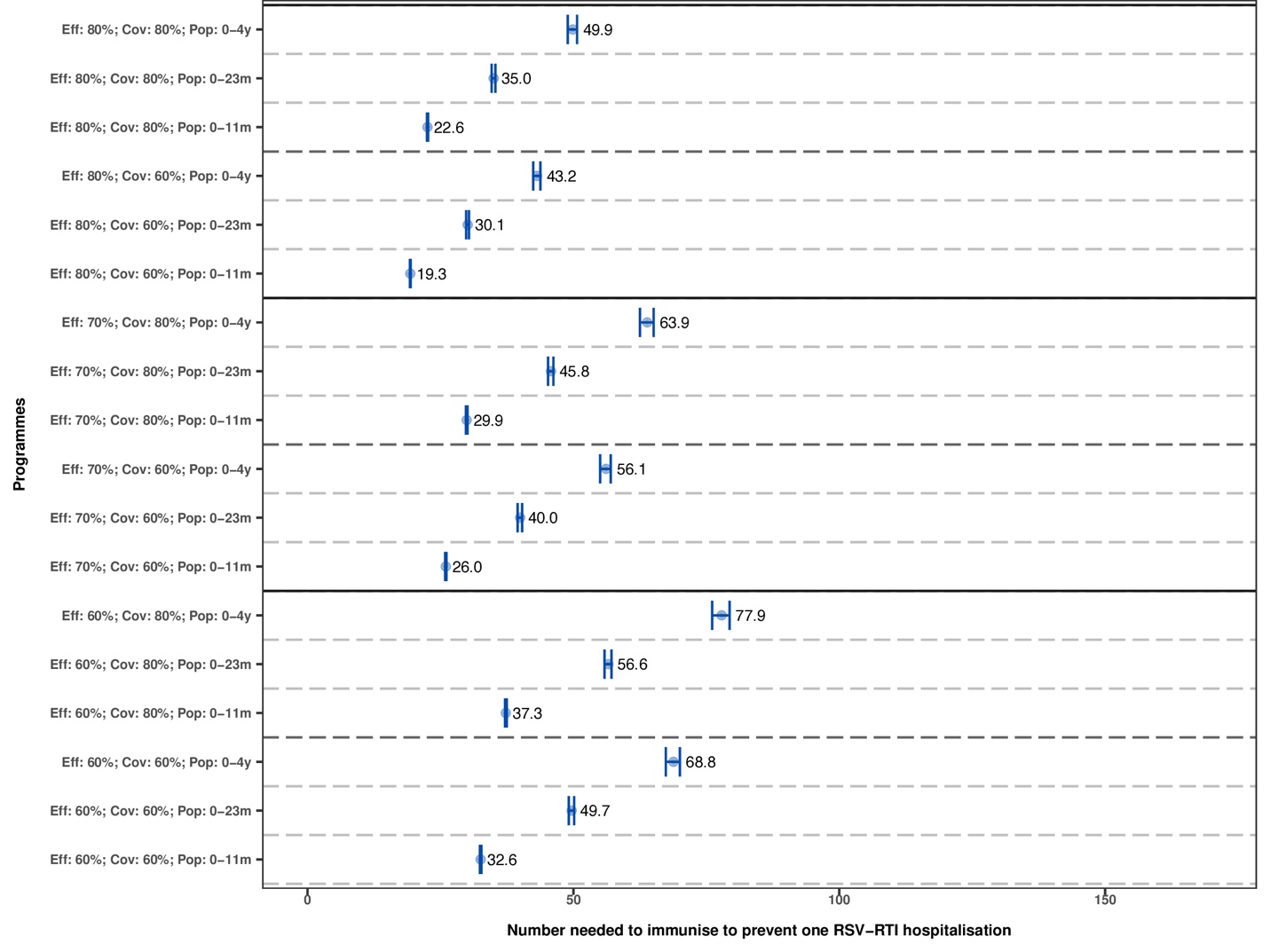


# Figure S10. The number of people need to be immunised to prevent one RSV hospitalisation under best-fitting model.

This figure illustrates the number of people need to be vaccinated to avert one hospitalisation case based on varying immunisation parameters. Abbreviations: Eff, efficacy of immunisation programme; Cov, coverage of immunisation programme; Pop, eligible population. The susceptibility coefficient for 0–4 years old, 5–59 years old and 60 years and above is 1.0, 0.38 and 0.38.


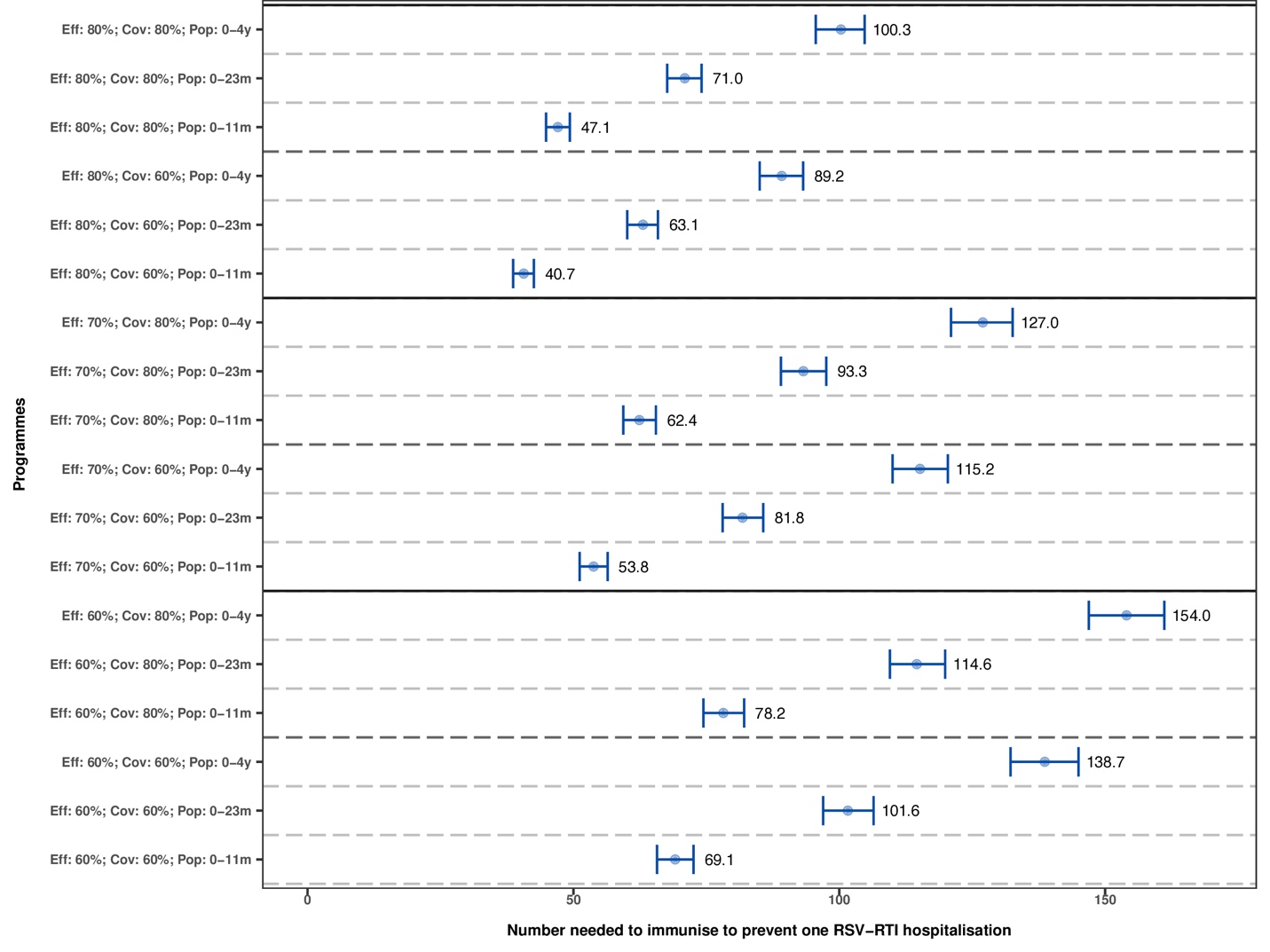


# Figure S11. The number of people need to be immunised to prevent one RSV hospitalisation under the base model with infection-hospitalisation ratios drived from best-fitting model.

This figure illustrates the number of people need to be vaccinated to avert one hospitalisation case based on varying immunisation parameters. Abbreviations: Eff, efficacy of immunisation programme; Cov, coverage of immunisation programme; Pop, eligible population. The susceptibility coefficient for 0–4 years old, 5–59 years old and 60 years and above is 1.0, 1.0 and 1.0.

# Table S1. Hospitalisation rate from previously research.

| Age | RSV-RTI hospital admissions | Population | RSV-RTI hospital admission rate per 1000 |
| --- | --- | --- | --- |
| 0–2 months | 858 | 14391 | 59.6206 |
| 3–5 months | 645 | 14391 | 44.81968 |
| 6–11 months | 699 | 28783 | 24.28517 |
| 12–35 months | 1074 | 116903 | 9.1871 |
| 3–4 years | 321 | 117619 | 2.72915 |
| 5–17 years | 314 | 744519 | 0.42175 |
| 18–64 years | 640 | 3352991 | 0.19087 |
| 65–74 years | 480 | 518497 | 0.92575 |
| 75 years and  above | 1843 | 424578 | 4.340780728 |

Age-specific hospitalisation rates from previous research were mapped to the closest age groups in this study. Specifically, the rate for ages 12–35 months was applied to the 12–23 months group; 3–4 years to 2–4 years; 5–17 years to 5–19 years; 18–64 years to 20–59 years; and the average rate for 65–74 years was applied to the group aged 60 years and above.

# Table S2. Estimated infection rate for infant and log-likelihood under different combination of age-specific parameters.

| ID | Age-specific susceptibility for 5–59 years | Age-specific susceptibility for 60 years and above | Infection rate | Log-likelihood |
| --- | --- | --- | --- | --- |
| GS001 | 0.4 | 0.2 | 50.49 | -38.069 |
| GS002 | 0.42 | 0.2 | 48.92 | -92.6797 |
| GS003 | 0.44 | 0.2 | 47.64 | -157.7888 |
| GS004 | 0.46 | 0.2 | 46.21 | -254.7249 |
| GS005 | 0.48 | 0.2 | 45 | -357.9628 |
| GS006 | 0.5 | 0.2 | 43.75 | -484.9627 |
| GS007 | 0.52 | 0.2 | 42.61 | -621.0422 |
| GS008 | 0.54 | 0.2 | 41.07 | -835.0861 |
| GS009 | 0.56 | 0.2 | 40.21 | -972.0873 |
| GS010 | 0.58 | 0.2 | 39.18 | -1152.2345 |
| GS011 | 0.4 | 0.22 | 50.44 | -39.4749 |
| GS012 | 0.42 | 0.22 | 49.38 | -73.7624 |
| GS013 | 0.44 | 0.22 | 47.59 | -160.782 |
| GS014 | 0.46 | 0.22 | 46.27 | -250.1233 |
| GS015 | 0.48 | 0.22 | 44.92 | -365.0266 |
| GS016 | 0.5 | 0.22 | 43.87 | -471.1221 |
| GS017 | 0.52 | 0.22 | 42.66 | -613.7487 |
| GS018 | 0.54 | 0.22 | 41.27 | -805.0674 |
| GS019 | 0.56 | 0.22 | 40.17 | -978.7797 |
| GS020 | 0.58 | 0.22 | 39.21 | -1146.7773 |
| GS021 | 0.4 | 0.24 | 51.06 | -24.9475 |
| GS022 | 0.42 | 0.24 | 49.62 | -64.8914 |
| GS023 | 0.44 | 0.24 | 47.93 | -141.6987 |
| GS024 | 0.46 | 0.24 | 46.5 | -233.4418 |
| GS025 | 0.48 | 0.24 | 45.58 | -306.2734 |
| GS026 | 0.5 | 0.24 | 44.04 | -453.7365 |
| GS027 | 0.52 | 0.24 | 42.67 | -613.3853 |
| GS028 | 0.54 | 0.24 | 41.64 | -750.6473 |
| GS029 | 0.56 | 0.24 | 40.52 | -921.5393 |
| GS030 | 0.58 | 0.24 | 39.34 | -1124.4118 |
| GS031 | 0.4 | 0.26 | 50.92 | -28.0196 |
| GS032 | 0.42 | 0.26 | 49.56 | -66.9874 |
| GS033 | 0.44 | 0.26 | 48.02 | -136.6701 |
| GS034 | 0.46 | 0.26 | 46.69 | -219.3551 |
| GS035 | 0.48 | 0.26 | 45.5 | -313.2388 |
| GS036 | 0.5 | 0.26 | 44.05 | -451.8054 |
| GS037 | 0.52 | 0.26 | 42.95 | -577.7959 |
| GS038 | 0.54 | 0.26 | 41.64 | -751.1927 |
| GS039 | 0.56 | 0.26 | 40.47 | -928.9486 |
| GS040 | 0.58 | 0.26 | 39.48 | -1098.8859 |
| GS041 | 0.4 | 0.28 | 51 | -26.3163 |
| GS042 | 0.42 | 0.28 | 50.01 | -51.7623 |
| GS043 | 0.44 | 0.28 | 48.05 | -135.0164 |
| GS044 | 0.46 | 0.28 | 46.95 | -201.7394 |
| GS045 | 0.48 | 0.28 | 45.51 | -312.1675 |
| GS046 | 0.5 | 0.28 | 44.24 | -431.9597 |
| GS047 | 0.52 | 0.28 | 43.06 | -564.32 |
| GS048 | 0.54 | 0.28 | 41.75 | -736.0109 |
| GS049 | 0.56 | 0.28 | 40.65 | -901.1946 |
| GS050 | 0.58 | 0.28 | 39.61 | -1074.3915 |
| GS051 | 0.4 | 0.3 | 51.24 | -21.6108 |
| GS052 | 0.42 | 0.3 | 49.94 | -54.1829 |
| GS053 | 0.44 | 0.3 | 48.33 | -120.2023 |
| GS054 | 0.46 | 0.3 | 47.17 | -186.7279 |
| GS055 | 0.48 | 0.3 | 45.77 | -289.7582 |
| GS056 | 0.5 | 0.3 | 44.38 | -418.4282 |
| GS057 | 0.52 | 0.3 | 43.2 | -547.28 |
| GS058 | 0.54 | 0.3 | 41.92 | -711.9932 |
| GS059 | 0.56 | 0.3 | 40.75 | -885.3248 |
| GS060 | 0.58 | 0.3 | 39.78 | -1044.9082 |
| GS061 | 0.4 | 0.32 | 51.53 | -16.8293 |
| GS062 | 0.42 | 0.32 | 49.6 | -65.6772 |
| GS063 | 0.44 | 0.32 | 48.45 | -114.254 |
| GS064 | 0.46 | 0.32 | 47.16 | -187.5931 |
| GS065 | 0.48 | 0.32 | 45.73 | -293.2425 |
| GS066 | 0.5 | 0.32 | 44.25 | -431.1686 |
| GS067 | 0.52 | 0.32 | 43.17 | -551.3694 |
| GS068 | 0.54 | 0.32 | 41.84 | -723.0098 |
| GS069 | 0.56 | 0.32 | 40.95 | -853.2058 |
| GS070 | 0.58 | 0.32 | 39.86 | -1031.1765 |
| GS071 | 0.4 | 0.34 | 51.67 | -14.9452 |
| GS072 | 0.42 | 0.34 | 50.02 | -51.5833 |
| GS073 | 0.44 | 0.34 | 48.42 | -115.6662 |
| GS074 | 0.46 | 0.34 | 46.99 | -198.9153 |
| GS075 | 0.48 | 0.34 | 45.94 | -275.7476 |
| GS076 | 0.5 | 0.34 | 44.55 | -400.3878 |
| GS077 | 0.52 | 0.34 | 43.24 | -543.3211 |
| GS078 | 0.54 | 0.34 | 42.04 | -694.9378 |
| GS079 | 0.56 | 0.34 | 40.87 | -866.6511 |
| GS080 | 0.58 | 0.34 | 39.84 | -1035.4662 |
| GS081 | 0.4 | 0.36 | 51.65 | -15.1006 |
| GS082 | 0.42 | 0.36 | 50.09 | -49.4355 |
| GS083 | 0.44 | 0.36 | 48.62 | -106.0744 |
| GS084 | 0.46 | 0.36 | 47.32 | -177.5985 |
| GS085 | 0.48 | 0.36 | 45.92 | -277.9785 |
| GS086 | 0.5 | 0.36 | 44.6 | -395.76 |
| GS087 | 0.52 | 0.36 | 43.32 | -533.2172 |
| GS088 | 0.54 | 0.36 | 42.17 | -677.3543 |
| GS089 | 0.56 | 0.36 | 41.15 | -823.3746 |
| GS090 | 0.58 | 0.36 | 39.98 | -1011.0288 |
| GS091 | 0.4 | 0.38 | 51.37 | -19.321 |
| GS092 | 0.42 | 0.38 | 50.03 | -51.0783 |
| GS093 | 0.44 | 0.38 | 48.28 | -122.922 |
| GS094 | 0.46 | 0.38 | 47.37 | -174.1961 |
| GS095 | 0.48 | 0.38 | 45.96 | -274.9807 |
| GS096 | 0.5 | 0.38 | 44.81 | -375.6321 |
| GS097 | 0.52 | 0.38 | 43.39 | -525.8771 |
| GS098 | 0.54 | 0.38 | 42.13 | -683.401 |
| GS099 | 0.56 | 0.38 | 41.13 | -826.6901 |
| GS100 | 0.58 | 0.38 | 40.04 | -1001.4497 |
| GS101 | 0.34 | 0.2 | 54.93 | -24.0316 |
| GS102 | 0.36 | 0.2 | 53.08 | -7.535 |
| GS103 | 0.38 | 0.2 | 51.98 | -11.4422 |
| GS104 | 0.34 | 0.22 | 54.97 | -24.742 |
| GS105 | 0.36 | 0.22 | 53.91 | -10.7407 |
| GS106 | 0.38 | 0.22 | 51.89 | -12.1976 |
| GS107 | 0.34 | 0.24 | 55.07 | -26.6329 |
| GS108 | 0.36 | 0.24 | 53.82 | -9.9267 |
| GS109 | 0.38 | 0.24 | 52.06 | -10.3889 |
| GS110 | 0.34 | 0.26 | 55.25 | -30.2719 |
| GS111 | 0.36 | 0.26 | 54.01 | -11.3023 |
| GS112 | 0.38 | 0.26 | 52.73 | -7.6517 |
| GS113 | 0.34 | 0.28 | 55.63 | -39.2334 |
| GS114 | 0.36 | 0.28 | 54.26 | -13.8024 |
| GS115 | 0.38 | 0.28 | 52.42 | -8.8605 |
| GS116 | 0.34 | 0.3 | 55.68 | -40.356 |
| GS117 | 0.36 | 0.3 | 54.1 | -11.9156 |
| GS118 | 0.38 | 0.3 | 52.57 | -8.0537 |
| GS119 | 0.34 | 0.32 | 55.7 | -40.9119 |
| GS120 | 0.36 | 0.32 | 54.2 | -13.1188 |
| GS121 | 0.38 | 0.32 | 52.59 | -7.9506 |
| GS122 | 0.34 | 0.34 | 56.03 | -49.6857 |
| GS123 | 0.36 | 0.34 | 54.59 | -18.2739 |
| GS124 | 0.38 | 0.34 | 52.81 | -7.713 |
| GS125 | 0.34 | 0.36 | 55.76 | -42.2835 |
| GS126 | 0.36 | 0.36 | 54.27 | -13.9194 |
| GS127 | 0.38 | 0.36 | 52.48 | -7.9979 |
| GS128 | 0.34 | 0.38 | 55.82 | -43.8823 |
| GS129 | 0.36 | 0.38 | 54.37 | -15.1614 |
| GS130 | 0.38 | 0.38 | 52.99 | -7.4883 |
| GS131 | 0.34 | 0.4 | 55.62 | -38.9497 |
| GS132 | 0.36 | 0.4 | 54.46 | -16.3446 |
| GS133 | 0.38 | 0.4 | 52.74 | -7.7241 |
| GS134 | 0.42 | 0.4 | 50.14 | -47.8528 |
| GS135 | 0.44 | 0.4 | 48.51 | -111.2738 |
| GS136 | 0.46 | 0.4 | 47.24 | -182.6796 |
| GS137 | 0.48 | 0.4 | 46.08 | -264.8941 |
| GS138 | 0.5 | 0.4 | 44.68 | -387.7116 |
| GS139 | 0.52 | 0.4 | 43.44 | -519.6952 |
| GS140 | 0.54 | 0.4 | 42.37 | -651.8487 |
| GS141 | 0.56 | 0.4 | 41.11 | -828.5676 |
| GS142 | 0.58 | 0.4 | 40.12 | -987.2644 |
| GS143 | 0.3 | 0.2 | 52.69 | -11.0633 |
| GS144 | 0.32 | 0.2 | 56.07 | -50.7464 |
| GS145 | 0.6 | 0.2 | 38.14 | -1354.3726 |
| GS146 | 0.3 | 0.22 | 53.36 | -11.1136 |
| GS147 | 0.32 | 0.22 | 56.28 | -56.9588 |
| GS148 | 0.6 | 0.22 | 38.25 | -1332.6677 |
| GS149 | 0.3 | 0.24 | 54.02 | -11.1685 |
| GS150 | 0.32 | 0.24 | 56.49 | -63.7436 |
| GS151 | 0.6 | 0.24 | 38.34 | -1313.2807 |
| GS152 | 0.3 | 0.26 | 54.67 | -19.4817 |
| GS153 | 0.32 | 0.26 | 56.53 | -64.8931 |
| GS154 | 0.6 | 0.26 | 38.47 | -1289.1524 |
| GS155 | 0.3 | 0.28 | 55.35 | -32.4318 |
| GS156 | 0.32 | 0.28 | 56.72 | -71.3597 |
| GS157 | 0.6 | 0.28 | 38.57 | -1269.0381 |
| GS158 | 0.3 | 0.3 | 56.06 | -50.719 |
| GS159 | 0.32 | 0.3 | 57.02 | -82.2252 |
| GS160 | 0.6 | 0.3 | 38.57 | -1269.0381 |
| GS161 | 0.3 | 0.32 | 56.63 | -68.2783 |
| GS162 | 0.32 | 0.32 | 57.19 | -88.3398 |
| GS163 | 0.6 | 0.32 | 38.67 | -1249.3041 |
| GS164 | 0.3 | 0.34 | 57.18 | -88.1568 |
| GS165 | 0.32 | 0.34 | 57.12 | -85.831 |
| GS166 | 0.6 | 0.34 | 38.76 | -1231.7797 |
| GS167 | 0.3 | 0.36 | 57.72 | -109.8539 |
| GS168 | 0.32 | 0.36 | 57.43 | -97.6997 |
| GS169 | 0.6 | 0.36 | 38.8 | -1225.1786 |
| GS170 | 0.3 | 0.38 | 58.11 | -127.2097 |
| GS171 | 0.32 | 0.38 | 56.88 | -80.5044 |
| GS172 | 0.6 | 0.38 | 39.12 | -1164.6156 |
| GS173 | 0.3 | 0.4 | 58.33 | -137.4764 |
| GS174 | 0.32 | 0.4 | 57.06 | -83.5013 |
| GS175 | 0.6 | 0.4 | 38.94 | -1196.6453 |
| GS176 | 0.4 | 0.4 | 51.49 | -17.5374 |

# Table S3. Parameter estimates from different models.

| Parameters | Description | Base model | Best-fitting model |
| --- | --- | --- | --- |
| $\delta^{0-4y}$ | Age-specific susceptibility for 0–4 years | 1.0 | 1.0 |
| $\delta^{5-59y}$ | Age-specific susceptibility for 5–59 years | 1.0 | 0.38 |
| $\delta^{60+y}$ | Age-specific susceptibility for 60 years and above | 1.0 | 0.38 |
| $\beta_{base}$ | Average transmission coefficient | 0.36 (0.35, 0.36) | 0.85 (0.84, 0.86) |
| $\beta_{seasonal}$ | Amplitude of the transmission rate | 3.55 (3.10, 4.05) | 9.71 (8.53, 10.85) |
| $\phi$ | Phase shift of the transmission rate | 30.62 (28.71, 32.63) | 31.04 (28.84, 32.68) |
| $p^{0-2m}$ | IHR for 0–2 months | 0.20 (0.19, 0.21) | 0.089 (0.086, 0.092) |
| $p^{3-5m}$ | IHR for 3–5 months | 0.16 (0.15, 0.17) | 0.076 (0.074, 0.079) |
| $p^{6-11m}$ | IHR for 6–11 months | 0.11 (0.10, 0.12) | 0.053 (0.051, 0.055) |
| $p^{12-23m}$ | IHR for 12–23 months | 0.022 (0.021, 0.023) | 0.013 (0.012, 0.013) |
| $p^{2-4y}$ | IHR for 2–4 years | 0.0074 (0.0071, 0.0076) | 0.0045 (0.0044, 0.0046) |
| $p^{5-19y}$ | IHR for 5–19 years | 0.00082 (0.00080, 0.00084) | 0.00091 (0.00089, 0.00094) |
| $p^{20-59y}$ | IHR for 20–59 years | 0.00064 (0.00062, 0.00067) | 0.00072 (0.00069, 0.00075) |
| $p^{60-64y}$ | IHR for 60–64 years | 0.003 (0.0029, 0.0032) | 0.0035 (0.0033, 0.0036) |
| $p^{65-69y}$ | IHR for 65–69 years | 0.0031 (0.0029, 0.0032) | 0.0036 (0.0034, 0.0038) |
| $p^{70-74y}$ | IHR for 70–74 years | 0.0038 (0.0036, 0.0039) | 0.0044 (0.0042, 0.0047) |
| $p^{75+y}$ | IHR for 75 years and above | 0.028 (0.027, 0.029) | 0.033 (0.032, 0.035) |
